# Socioeconomic Determinants of Health and Their Unequal Distribution in Poland

**DOI:** 10.3390/ijerph182010856

**Published:** 2021-10-15

**Authors:** Justyna Rój, Maciej Jankowiak

**Affiliations:** 1 Department of Operational Research and Mathematical Economics, The Poznań University of Economics and Business, Al. Niepodległości 10, 61-875 Poznań, Poland; 2 Department of Organisation and Healthcare Management, Poznań University of Medical Sciences, ul. Przybyszewskiego 39, 60-356 Poznań, Poland

**Keywords:** equity, health, socioeconomic, mortality rate, regional policy, two-stage nested Theil index, Herfindahl-Hirschman Index, regression analyses

## Abstract

The purpose of this study is to identify inequities in the distribution of socioeconomic determinants of health within Poland and their impact on the health status of Poles, as measured by mortality rate. We hypothesised that (1) there are inequities in the socioeconomic characteristics within geographically defined population groups and (2) some socioeconomic determinants of health have a particularly strong impact on the health status of Poles. Poland is administratively divided into three levels: voivodeships, powiats and gminas. We used a dataset covering all 380 powiats in Poland for the year 2018. We employed a two-stage nested Theil index and Herfindahl–Hirschman Index. In order to identify which of these determinants has the strongest impact on health, we conducted a regression analysis. The study revealed some inequities in the distribution of socioeconomic determinants of health. The mortality rate can be partly understood from variations within voivodeships in the distribution of health determinants. Important national inequalities were found in the case of two determinants, which simultaneously proved their significant impact on Poles’ health in the regression analysis. Thus, type of employment and access to modern infrastructure should be of particular concern for public authorities.

## 1. Introduction

Health is important for a variety reasons, in particular for individuals’ well-being and ability to pursue different life plans [1]. However, health is quite complex as, according to the World Health Organization, it is “a state of complete physical, mental and social well-being” [2]. The complexity of health is reflected by the production function of health, which was first described by Auster, Leveson and Sarachek (1969) [3]. They examined health (measured by mortality rate) as a function of both medical care and environmental variables [3]. Thus, the health production function describes “the relationship between combinations of medical and non-medical inputs and the resulting output” [4]. Many researchers have used this concept in their studies, but they have also started to employ different variables to explain health status [5]. Thus, many empirical analyses have considered income as one of the main determinants of health, followed by education, food quality, health expenditure, social protection, early childhood development, food insecurity, unemployment and job insecurity, working life conditions, housing, basic amenities and environment, social inclusion and non-discrimination, structural conflict and access to affordable health services of a decent quality [6]. Table 1—based on selected existing studies—shows the diversity of the determinants of health and health outcome measures in use. These analysed variables are actually the conditions in which people are born, grow up, live, work and age; in the literature they are described as the socioeconomic determinants of health [7,8].

The socioeconomic determinants of health can be grouped into five key areas or domains [9]: (1) economic stability; (2) education access and quality; (3) neighbourhood and built environment; (4) social and community context; and (5) health care access and quality.

The first group of socioeconomic determinants applies to economic stability and social status. It includes working conditions, which can provide financial security, social or employment status, social relations, personal development, self-esteem and protection from physical and psychosocial hazards [9]. Unemployment can cause psychosocial stress and can thus have a strong impact on physical and mental health and well-being [10]. In addition, as children age, the relationship between household income and their health becomes more pronounced [11].

Education (access and quality) is the second group of socioeconomic determinants. It includes such factors as education attainment in general, early childhood education and development, high school graduation, enrolment in higher education, language and literacy [7,12]. The positive relationship between more years of education and better health is one of the most fundamental connections in health economics, which originates from Grossman’s health demand model [13]. In addition, it is an empirically well-documented pattern in the literature [14,15,16]. Education improves health, as it increases knowledge, skills, reasoning, effectiveness and other abilities as well as enhancing a sense of personal control that can enable a healthy lifestyle [17,18,19].

The third group of socioeconomic determinants is the quality of one’s neighbourhood and built environment (physical environment). This group includes housing and shelter, transportation and roads, food, air and water quality, sanitation, neighbourhood crime and violence, safety, parks, walkability, and local geography and communities [10]. Homelessness and overcrowding are risk factors of physical and mental health [8,10]. The current model of urbanisation and the lack of balance between rural and urban areas pose many environmental challenges, especially those connected with climate change [12]. Good quality housing requires appropriate heating. Housing with insufficient heat is linked with a number of cardiovascular and respiratory morbidity outcomes, as well as increased incidence of psychological conditions such as depression. Heating also determines the hygrothermal conditions in the house. Inappropriate hygrothermal conditions may favour the proliferation of mites and asthma [20,21,22,23]. By having access to energy, people can have vital health determinants in the house, such as heating and the means to prepare nutritious food [24].

In addition, many empirical studies have shown that people’s perception of the built environment may directly influence mental stress, which has an impact on their well-being and overall health. It can also indirectly influence health through health-related behaviours such as social contact or physical activity [25]. Additionally, active transportation can increase physical activity and thus health [26,27].

The next group of socioeconomic determinants—social and community context (social support networks)—includes community engagement, social integration, support systems, the beliefs of the family and community, customs and traditions, civic participation and discrimination. Having greater support from families, friends and communities is linked to better health [9,28].

The final group of determinants applies to access to and quality of health care, which means access to primary health care, health insurance coverage, health literacy, quality of care and utilisation of health care; these elements are integral in the prevention and treatment of disease and generally influence health [10,29,30].

By forming the conditions of daily life, these socioeconomic determinants influence people’s opportunities to be and stay healthy [31]. Moreover, these socioeconomic determinants of health have varied across space and time over the last few decades. Since they are also under the influence of different socioeconomic processes, some of them are more significant or fundamental than others. For example, the process of urbanisation has improved standards of living, positively influencing health, but negatively impacting lifestyles and lifestyle-related factors, including physical inactivity and unhealthy diets [32]. In addition, deindustrialisation has led to greater levels of socioeconomic deprivation (and associated factors) and has resulted in relatively poor health status among people in deindustrialised areas [33]. The global financial crisis has caused a dramatic transformation of employment in the weakest economies of the Eurozone. The deterioration of working conditions, low pay and periods of prolonged unemployment for most of the working population—especially women—have been observed [34]. Financialisation has exerted significant effects on many aspects of our daily life, such as consumption patterns, housing affordability, employment structure and social conditions, which are relevant to health. Generally, it has contributed to increased income inequalities through different channels [35].

The uneven distribution of socioeconomic determinants contributes to intergroup differences in health outcomes, both within and between societies [31], which is a major obstacle in achieving health justice [36]. Thus, ensuring health equity requires the elimination of unfair and avoidable differences in health among population groups, which are defined economically, socially, geographically or demographically [37,38].

Thus, the unequal allocation of power as well as resources, which appears in unequal social, economic and physical conditions, is recognised as one of the main root causes of health inequity [7,39]. It is mainly derived from the existence of inequalities in other areas of life, such as economic, political or social spheres [40]. This is the result of decision-making processes, policies, social norms and structures, which exist at all levels in society; therefore, effective interventions are required in all sectors [31,41]. Thus, the socioeconomic determinants are modifiable and can be influenced by social, economic and political processes, historical and contemporary policies, law, investment, culture and norms [7]. As socioeconomic determinants affect how people experience the world and the choices they make, bringing about a reduction in their distribution inequities is an important challenge for health policies [42,43].

Therefore, a burgeoning volume of research is focused on the social, economic and environmental determinants of health and their impact on health outcomes, as well as identifying these determinants as the main root cause of many health inequities. Many studies—which have covered different world populations and various ranges of socioeconomic determinants (Table 1)—have shown that people from lower socioeconomic groups have shorter lives and more often suffer from health problems, while people with a quality education, stable employment, safe homes and neighbourhoods and access to preventive services tend to be healthier throughout their lives.

Previous research in the area of socioeconomic inequities in the health of Poles has primarily compared Poland with other countries [44,45]. There are other studies having a limited context, such as those focusing on economic status and gender [46]; education, marital status, employment status or place of residence and their impact on mortality among working-age people [47]; the social determinants of the self-rated health of Polish women and men [48]; and the relation between expenses for health and healthy life expectancy [49]. There have also been a few studies on the socioeconomic determinants of the health of rural inhabitants [50,51].

As reducing health inequities is treated as a matter of social justice and is thus a kind of ethical imperative, the Commission on Social Determinants of Health called on the WHO and all governments “to lead global action on the social determinants of health with the aim of achieving health equity” [12]. In the Polish health care system, both health and equity are important values [52,53], as determined from the WHO constitution and strategy [2] as well as from Article 68 of the Polish Constitution [54]. In addition, one of the strategic objectives of the Polish national health policy, as formulated in the National Health Programme [55], is the elimination of geographical and social inequalities in health.

Therefore, in this study we focus on identifying inequities in the distribution of socioeconomic determinants of health within Poland and the impact of socioeconomic determinants on the health status of Poles, as measured by mortality rate. The aim of this study is to measure the level of inequities in the distribution of socioeconomic determinants of health between geographically defined groups of people in Poland. The hypotheses are as follows:

(1) There are inequities in the socioeconomic characteristics within geographically defined population groups.
(2) Some socioeconomic determinants of health have a particularly strong impact on the health status of Poles.

In order to verify these hypotheses, we used the database of Statistics Poland [56], which determined the final range of socioeconomic variables and the year of research (2018) adopted for the study. Therefore, it was possible to derive the data at the *powiat* level—the second (out of three) administrative level in Poland—which made it possible to identify the sources of geographical inequities.

As it was conducted on the powiat level, our research fills an existing gap by providing more specific information on the spatial diversity of the Polish population in terms of the socioeconomic determinants of health. The novelty of this research also arises from it being the first time the two-stage nested Theil decomposition method is utilised in the context of the Polish population, allowing national inequity to be decomposed to macro-regions by comparing inequities between voivodeships and within voivodeships.

## 2. Materials and Methods

Poland is administratively divided into three levels, including 16 voivodeships (provinces), 380 powiats (including 66 cities with powiat status) and 2478 gminas (2018). Each voivodeship consists of powiats, and each powiat consists of gminas.

According to the Nomenclature of Territorial Units for Statistics (NUTS), Poland is divided into 7 macro-regions (NUTS 1), then 17 regions (NUTS 2) and 73 subregions (NUTS 3). Each macro-region consists of regions, and each region consists of subregions.

Voivodeships are conterminous with regions (NUTS 2), with one exception—Mazovian voivodeship—which is split into two NUTS 2 units (regions): Warsaw—capital and Masovian—regional. Thus, all (16) voivodeships can be classified into 7 macro-regions (NUTS 1). The above relations between macro-regions, regions and voivodeships in Poland are presented in the Table 2. Individual macro-regions reflect the economic and social development of various regions in Poland (Supplementary Material).

Thus, the level of unit data is the level of powiats for the purpose of analysing the inequity at the level of macro-regions and then the 16 voivodeships. Data were derived from the Statistics Poland database for 2018 [56]. The scope of the obtained data covered 380 powiats in Poland—i.e., all of them—for the year 2018. Including all the powiats in Poland in the study made it possible to obtain representativeness in the research and the results.

The range of variables and their measures (Table 3), i.e., socioeconomic determinants, were selected based on the analysis of previous studies ([18,19,20,21,22,23,24,25,26,27,28,29] and Table 1) and then determined by the availability of data. They were grouped into economic, education, employment, demography and built environment categories.

First, we determined the descriptive statistics. Analysis of the average and median of the analysed health determinants (Table 4) suggests that in case of most of them (14 out of 17—IN, EDE, EDJH, EA, EI, ES, EF, UR, FR, OR, GS, F, GR, DIS), more than 50% of powiat values had levels lower than the average. Based on the standard deviation and variation, it can be found that the IN variable is characterized by a high level of dispersion.

For the purposes of examining the distribution of the socioeconomic variables in Poland and to determine the drivers of inequity, the Theil index was employed. It was developed by Theil in 1967 and is widely used to measure spatial inequality [88]. The Theil index ranges between 0 and ∞, where zero represents an equal distribution and any higher value represents a higher level of disproportion.

Other commonly used methods to measure the level of inequity in the context of health and health care are the Gini index [38,52,59] and concentration index [57,58,60]. Compared with the Gini coefficient, when estimating regional differences, the Theil index allows sub-groups to be broken down within the context of larger groups. Thus, it is possible to analyse their contribution to the total differences and to identify the main sources of the overall differences [89]. This is an important property of the Theil index measure, as this additive decomposability implies that the aggregate inequality measure can be broken down into inequality within and between any defined population subgroups [90]. The main pitfall of the Theil index is that its values are not always comparable across completely different units, as in case of different nations. If the number and size of groups differ, then limit of the index will differ [91].

Since this article considered the three division scales of macro-region, voivodeship and powiat in Poland, it is more suitable to apply the two-stage nested Theil decomposition method as proposed by Takahiro Akita in 2003 [92]. This two-stage nested Theil index allows us to decompose the national overall inequality into between-macro-regions, between-voivodeships and within-voivodeships. Through such decomposition, the Theil index can comprehensively reflect the multi-scale inequality in the distribution of socioeconomic determinants, with each component explaining a part of overall inequality that is due to differences within and between voivodeships and between macro-regions.

The overall national inequality, *T*, of a particular socioeconomic variable distribution, based on the powiat level, can be measured using the following formula [92,93]:

(1) $$T=\sum_{i}\sum_{j}\sum_{k}\left(\frac{L_{ijk}}{L}\right)\log\left(\frac{L_{ijk}/L}{P_{ijk}/P}\right)$$

where

*L_ijk_*—the particular socioeconomic determinant of health in powiat *k* in voivodeship *j* in macro-region *i*;
*P_ijk_*—the total population (or subpopulation, in case of EDE, EDJH, the children in the appropriate range of age were used as the subpopulation; in case of EA, EI, ES, EF, UR, the working age population was used as the subpopulation; where appropriate) in powiat *k* in voivodeship *j* in macro-region *i*;
*L*—the overall national socioeconomic determinant of health;
*P*—the overall national population (or subpopulation^1^, where appropriate).

Then, *T_ij_* is defined as the inequity in voivodeship *j* in macro-region *i.*

(2) $$T_{ij}=\sum_{k}\left(\frac{L_{ijk}}{L_{ij}}\right)\log\left(\frac{L_{ijk}/L_{ij}}{P_{ijk}/P_{ij}}\right)$$

*T_i_*, as the inequality in macro-region *i*, can be decomposed using the following equation:

(3) $$T_{i}=\sum_{j}\sum_{k}\left(\frac{L_{ijk}}{L_{i}}\right)\log\left(\frac{L_{ijk}/L_{i}}{P_{ijk}/P_{i}}\right)=\sum_{j}\left(\frac{L_{ij}}{L_{i}}\right)T_{ij}+\sum_{j}\left(\frac{L_{ij}}{L_{i}}\right)\log\left(\frac{L_{ij}/L_{i}}{P_{ij}/P_{i}}\right)=T_{wi}+T_{pi}$$

where

*L_ij_*—the particular socioeconomic determinant of health in voivodeship *j* and in macro-region *i*;
*P_ij_*—the total national population (or subpopulation^1^, where appropriate) in voivodeship *j* and in macro-region *i*;
*L_i_*—the socioeconomic determinant of health in macro-region *i*;
*P_i_* —the total national population (or subpopulation^1^, where appropriate) in macro-region *i;*
*T_wi_*—measures within-voivodeship inequality;
*T_pi_*—measures between-voivodeships inequality.

By combining all of the above formulas, the overall national differences, *T*, can be expressed as follows, which is the final form of the two-stage nested Theil decomposition method equation:

(4) $$T=\sum_{i}\left(\frac{L_{i}}{L}\right)T_{i}+\sum_{i}\left(\frac{L_{i}}{L}\right)\log\left(\frac{L_{i}/L}{P_{i}/P}\right)=\sum_{i}\left(\frac{L_{i}}{L}\right)T_{i}+T_{BR}=\sum_{i}\left(\frac{L_{i}}{L}\right)\left(T_{wi}+T_{pi}\right)+T_{BR}=\;\sum_{i}\left(\frac{L_{i}}{L}\right)T_{wi}+\sum_{i}\left(\frac{L_{i}}{L}\right)T_{pi}+T_{BR}=T_{WP}+T_{BP}+T_{BR}$$

where

*T_WP_*—within-voivodeship component;
*T_BP_*—between-voivodeship component;
*T_BR_*—between-macro-region component.

For the purposes of assessing the level of inequity of the analysed variables, the Herfindahl-Hirschman Index (HHI) [94] was also employed, which allows us to identify the level of inequity in the distribution of the socioeconomic determinants of health. It is commonly used in economics, health services research and other disciplines [95].

The HHI can be defined as the sum of square of the shares of each variable in the overall sum of variables, and it is expressed by the following formula [94]:

HHI = ∑^n^*_i_*_= 1_ (*MS_i_*)^2^ (5)

where

*MS_i_*—the proportion of a percentage of a variable for *i*-powiats to a percentage of a variable in all powiats;
n—number of powiats in the macro-region.

The result is often multiplied then by 10,000; the distribution of variable is considered highly concentrated if the value of HHI is greater than 2500, moderately concentrated the HHI value is between 1500 and 2500, and unconcentrated if the HHI is between 100 and 1500 [96].

Then, multiple regression analysis was employed in order to identify the most significant determinants of health at the level of Polish powiats. Mortality rate was adopted as a measure of the health status of the population [97] and incorporated into the regression model as a dependent variable. The mean value of the dependent variable was 10.86, median 10.75, maximum 17.53, minimum 6.45, variance 2.33 and standard deviation 1.53. Distribution of the dependent variable was tested using the chi square test and was found to be normal. The Independent variables initially considered were the above-mentioned 17 determinants of health (see Table 3). In the first step of the analysis, two-sided correlations between each of independent variables and a dependent variable (the mortality rate) were assessed using the Spearman’s rank correlation coefficient. Calculated absolute values of the coefficient are presented in Table 5. As a cut-off point of a significant correlation, the coefficient absolute value of 0.1 was adopted. Six of the independent variables (EDE, EDJH, EF, WS, F, GR) reached the absolute value of the correlation coefficient of less than 0.1 and were excluded from further analysis. The remaining 11 independent variables were included in the preliminary regression model.

The second step of the regression analysis was development of the preliminary multiple linear regression model, containing the independent variables (IN, EA, EI, ES, UR, WAP, FR, OR, SS, GS, DIS) significantly correlated with the mortality rate. The general formula of the regression model is given below:

Y = a_1_X_1_ + a_2_X_2_ + … + a_n_X_n_ + B (6)

where: Y—the predicted value of the dependent variable; X_1_, X_2_, … X_n_—the independent variables; a_1_, a_2_, … a_n_—the regression coefficients (slopes) of the independent variables; B—the intercept.

The parameters (slopes and an intercept) of the preliminary model were established using the least squares estimation. For each of independent variables, a *p*-value was calculated employing the t-statistic. The significance level α = 0.05 was adopted. a *p*-value above 0.05 indicated statistically non-significant variables.

In the next step of the analysis, the preliminary model was refined. Four of the non-significant independent variables (IN, EI, ES, DIS) were excluded. The final model consisted of seven independent variables: EA, UR, WAP, FR, OR, SS, GS. The parameters of the final model (slopes and an intercept) were recalculated using the least squares approach, and t-statistics were employed for calculation of the independent variables’ *p*-values as well.

Additionally, the final model was tested with regard to statistical independence of the random errors with the use of the Durbin–Watson statistic. According to the D–W distribution tables, a value of the D–W statistic between 1.84 and 2.16 was adopted as an indicator of the absence of residual auto-correlations at a significance level α = 0.05, which means that there is no violation of independence of the random errors in the final regression model.

Calculations of the Theil index and the HHI were done using a free software spreadsheet. Calculation of the regression model was done using STATISTICA software (TIBCO Software Inc., Statistica version 13. (Palo Alto, CA, USA).

## 3. Results

The Theil index was employed to measure the nationwide equity of the distribution of socioeconomic variables in Poland and the contribution rate of each Polish voivodeship. The Theil index values shown in Table 6 indicate the existence of inequity in the distribution of such variables as GR, EF, F, EA, DIS, GS and ES. The values for these variables range from 0.1230–0.4644, while any value higher than 0 indicates some level of disproportion. In the case of the remaining variables, slight inequity can be observed, but the values are generally below 0.0684.

It can be concluded that at the national level, individual areas in Poland vary in importance in terms of the size of green areas and forests. Some variation in the area of employment structure can be observed, as there is a concentration of employment in finance and agriculture as well as a slight concentration in services. Poland is also characterised by inequity in adapting buildings for people with disabilities and supplying gas to homes.

Table 6 also contains results that show the contribution of three components of overall national inequality (T): the between-macro-region component (*TBR*), the between-voivodeship component (*TBP*) and the within-voivodeship component (*TWP*). In the case of the above-mentioned determinants (GR, EF, F, EA, DIS, GS and ERS), within-voivodeship inequity is largely responsible for their total unequal distribution, since the values of this component are generally higher than the other components (between-voivodeship inequity and between-macro-region inequity).

However, the within-voivodeship component constitutes the main component of overall national inequities for socioeconomic determinants other than the education variables (EDE and EDJH) (see Table 6). In the case of the education variables, the differentiation between voivodeships is mainly responsible for the slight inequities at the national level. Thus, the difference within voivodeships is the main factor leading to national differences in the socioeconomic determinants of health distribution, from a spatial perspective. The results confirm the hypothesis that there is an inequality of the distribution of the socioeconomic determinants of health and that it is caused by within-voivodeship differentiation.

**Figure 1:**
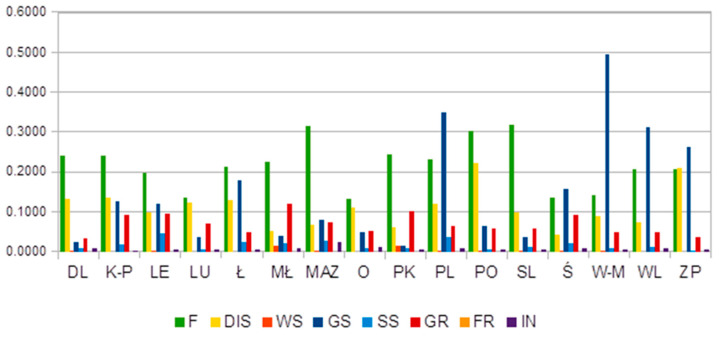
Within-voivodeship inequality. Source: Authors’ calculations.

As can be seen in Figure 1, the inequalities within voivodeships—i.e., between powiats—show different degrees of expansion, which led to the polarization of some of the socioeconomic determinants of health in 2018, such as forestation, gas supply, and the level of building adaptation for the disabled. High levels of inequity in the distribution of forests (F) were noted in the case of almost all voivodeships. Podlaskie (PL), Warmian-Masurian (W-M), Wielkopolska (WL) and Zachodniopomorskie (ZP) voivodeships presented some level of inequity in access to the gas supply system. Zachodniopomorskie (ZP) and Pomeranian (PO) voivodeships showed unequal distribution of cultural buildings adapted for the disabled.

**Figure 2:**
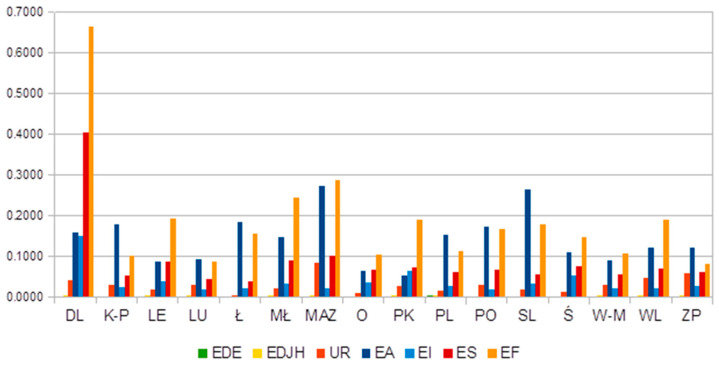
Within-voivodeship inequality. Source: Author’s calculation. Legend for Fig 1 and 2: (DL)—Lower Silesian, (K-P)—Kuyavian-Pomeranianranian, (LE)—Lubelskie, (LU)—Lubuskie, (Ł)—Łódż, (MŁ)—Lesser Poland, (O)—Opole, (PK)—Podkarpackie, (PL)—Podlaskie, (PO)—Pomeranian, (ŚL)—Silesian, (ŚW)—Świetokrzyskie, (W-M)—Warmian-Masurian, (WL)—Wielkopolska, (ZP)—Zachodniopomorskie, (MAZ)—Masovian, (W-stoł)—Warsaw—Capital, (MAZ_R)—Masovian—regional.

In the area of education and the labour market (Figure 2), it is noted that Lower Silesia (DL) presented the highest inequity in the distribution of employees in the finance and services areas between powiats. This could be caused by the high concentration of finance and services companies in Wroclaw—the capital of Lower Silesia. The results present high differentiation in Masovian (MAZ) and Silesian (ŚL) voivodeships, as they show some level of concentration of both agricultural and finance employees.

These results suggest that these identified differences may be a capital city effect and may represent an urban–rural divide, which has been observed in other areas researched in Poland [98,99]. Populations continue to expand in and around many capital cities and urban areas, as they are associated with (perceived) education and/or employment opportunities.

The HHI values for the socioeconomic determinants of health are presented in Table 7. The results present the concentration level of the above determinants and thus their distribution inequities.

Generally, the HHI values indicate a low level of variable concentration, as they are below 1500, especially in the case of the four macro-regions: south, north-west, north and east. If the HHI values are between 100 and 1500, then the particular feature is unconcentrated and is considered equally distributed.

There is one exception, as the distribution of employment in the financial sector (EF) demonstrated moderate concentration in the south and north macro-regions (the values were between 1500 and 2500). The south-west and central macro-regions were characterised by moderate concentrations of most variables, apart from the employment rate in finance, which showed a high level of concentration (the values were greater than 2500). This high level of EF concentration, and such inequities in its distribution between macro-regions, may be due to the existence of large, fast-growing economic and financial city centres, such as Wrocław (south-west) and Łódź (central).

The Masovian macro-region was characterised by moderate concentration in the case of old-age dependency ratio and employment rate in both agriculture and industry (the values ranged from 1500 to 2500) and a high level of inequity in terms of the remaining socioeconomic variables (the values were greater than 2500). This may be due to the fast-growing capital of Poland, Warsaw, which is surrounded by relatively few developed areas.

Initially, the 17 socioeconomic determinants of health listed in Table 3 were considered potential independent variables in a multiple linear regression analysis. Eleven of the determinants had a Spearman’s rank correlation coefficient of over 0.1 and had sufficient two-sided correlation with mortality rate and were thus used for the construction of the preliminary regression model. These were IN, EA, EI, ES, UR, WAP, FR, OR, SS, GS and DIS. The parameters (slopes and an intercept) of the preliminary model are presented in Table 8. An *r*^2^ value of 0.7906 indicates that this model describes about 79% of the variability in the mortality rate. Based on the *t* statistics, *p*-values for each independent variable were calculated; these are presented in Table 8.

Five of the independent variables (IN, EA, EI, ES and DIS) were statistically non-significant (a *p*-value above 0.05). Four of them (IN, EI, ES and DIS) were excluded from further analysis, so the final regression model was constructed with seven independent variables: EA, UR, WAP, FR, OR, SS and GS. The independent variable slopes and the intercept in the final model are presented in Table 8. The *r*^2^ value for the final model was 0.7220, which means that this model describes about 72% of the variability of the dependent variable (the mortality rate). In the final model, the *p*-value for all independent variables was below 0.05, indicating their statistical significance. A test of statistical independence of the random errors in the final model was done with the use of the Durbin–Watson statistic. The obtained value of the D–W statistic was 1.86, which means that at a significance level α = 0.05, no residual auto-correlations occurred, and thus the model has good diagnostic features.

## 4. Discussion

This study identified inequities in the distribution of socioeconomic determinants of health between geographically defined populations. It demonstrates that in Poland, as a result of their geographic status, people do not have equal opportunity to achieve their full health potential. The results confirmed that voivodeships are quite heterogeneous in terms of the distribution of the socioeconomic determinants of health. This implies the existence of inequities in the distribution of these determinants. The main risk factors of health inequity are observed in the conditions of the built environment and employment. Discrepancies in access to green areas, forests and the gas supply system, as well as the levels of employment in agriculture and finance, were found.

The selection of the powiat-level unit and the two-stage Theil index method allowed the identification of the level of national inequality in the distribution of the socioeconomic determinants of health in Poland. Moreover, these findings showed that this inequality across the country and in all macro-regions was decomposable and that the inequalities within voivodeships also represent an important part of national inequalities.

The research only partly confirmed the existence of a high disproportion between eastern Poland (colloquially called Poland B) and western Poland (Poland A), which was recognised in other studies [100]. The within-voivodeship component constitutes the main component of overall national inequities, while the between-voivodeship component is only responsible for some national inequity in the case of the education variables (EDE and EDJH). In addition, the most diversified voivodeships are located in both the east and west of Poland.

In addition, the values of HHI revealed that one macro-region in Poland—Masovia—was characterised by a high concentration of most of the health determinants. The Masovian macro-region contains the capital city, and such variation in the distribution of socioeconomic variables could be caused by different rates of development, leading to the growth of large centres and to increasingly poor surrounding areas, where there is no rapid economic growth [101].

Likewise, the south-west and central macro-regions were characterised by moderate concentrations of most variables. When we compare this result with those of Ucieklak-Jeż and Bem [51], who found that rural areas were homogeneous in terms of the analysed sociodemographic determinants of health, we suspect that the concentration of particular health determinants in urban areas could also have been the main reason for the variability among voivodeships or macro-regions. However, further research is required, as Ucieklak-Jeż and Bem [51] employed slightly different ranges of health determinants.

The low level of most socioeconomic variable concentrations, which was recognised in the case of the south, north-west and north macro-regions, can be explained by historical factors, which many publications have described as a mechanism that still maintains regional disparity in Poland [102,103]. The period of partitions, in particular, contributed to differences in socioeconomic development and social resources in individual regions in Poland that still exist today. This period contributed to the diversification of the behavioural characteristics of the population of the particular partitions.

Historical factors, therefore, caused regional differentiation in the importance awarded to local ties and economic attitudes [104], which, today, could favour equality or eliminate inequalities. The populations of the north and north-west macro-regions are characterised by greater entrepreneurship and a rational approach as well as greater economic activity, while the south of Poland is characterised by a high level of localism. As the northern and southern parts of Poland demonstrate similar levels of concentration in most socioeconomic determinants—lower than those of the other macro-regions—these results cannot be explained by variability in epidemiology [105]. Further research is required.

Based on correlation and multiple regression analysis, only some of the 17 socioeconomic determinants of health taken into consideration proved to have a significant impact on the mortality rate of the Polish population. Six of the independent variables (EDE, EDJH, EF, WS, F and GR) were weakly correlated with the mortality rate (the absolute values of the correlation coefficient were less than 0.1). The remaining 11 independent variables (IN, EA, EI, ES, UR, WAP, FR, OR, SS, GS and DIS) were had significant two-sided correlation with the mortality rate and were used in the preliminary regression model. This model showed good predictive value and explained about 79% of the variability in the mortality rate.

Nevertheless, not all independent variables in the preliminary model were statistically significant. The *p*-values calculated for four variables (IN, EI, ES and DIS) were much higher than the adopted α = 0.05 (0.482, 0.561, 0.685 and 0.553, respectively), which means that their potential ability to predict the mortality rate value is uncertain, despite being sufficiently correlated with the dependent variable.

In order to improve the regression model, these four variables were excluded from the final model. The final regression model consisted of seven independent variables: EA, UR, WAP, FR, OR, SS and GS. This model explained about 72% of the variability in the mortality rate, which is slightly less than in the preliminary model, but still represents good predictive value. For all independent variables, the *p*-values were less than 0.05, and their impact on the mortality rate could be perceived as being statistically significant. Based on the final regression model, four of the socioeconomic health determinants that were used had a positive influence on health status (they had negative regression slopes) and reduced the mortality rate: EA, FR, SS and GS. Three of the independent variables in the final model (UR, WAP and OR) had positive slopes. They increased the mortality rate and could be treated as risk factors of a deterioration in health status. In particular, the positive correlation between WAP and an increased mortality rate in the regression model requires further, focussed research. The within-country inequalities among these seven significant socioeconomic determinants of health identified in the Polish population could be particularly important to explain potential differences in health status at the powiat level. In the case of two significant determinants (EA and GS), the Theil index analysis indicated important national inequalities. These two determinants should not be interpreted too literally. EA can be treated more as an indicator of employment type (such as work in a healthy environment near one’s residence that lacks strong subordination in the chain of command), while GS can be seen as an estimator of infrastructure development (such as modern infrastructure with no significant negative impact on the human environment and health due to low dust emission). These results could mean that socioeconomic determinants related to employment type and infrastructure development should be of special concern in improving the health status equity of the Polish population, inducing actions to facilitate equal access to modern ecological infrastructure and to make an active workforce market policy that prioritizes equal access to jobs without consequences for workers’ health.

The study led to the identification of the voivodeships that suffer the most from internal differentiation in the distribution of the socioeconomic determinants of health. In the case of access to gas supply, the Podlaskie (PL), Warmian-Masurian (W-M), Wielkopolska (WL) and Zachodniopomorskie (ZP) voivodeships presented some level of inequity. In addition, high differentiation between the Masovian (MAZ) and Silesian (ŚL) voivodeships was observed, as concentrations of both agricultural and financial employees were found. Thus, studies similar to this one could be used to support policymakers and local governments as well as other stakeholders responsible for creating public regional policy.

Because many of these health differences are caused by decision-making processes, policies, social norms and structures, which exist at all levels in society, these results show the direction of changes that should be undertaken, especially in the Masovian macro-region. This study reveals that analysing variations in inequalities in the distribution of socioeconomic determinants of health within a country can help to identify entry points for policy. In this study, we proposed the two-stage nested Theil index to measure inequities in the socioeconomic determinants in Poland. This allowed analysis to be made at different statistical and administrative levels.

## 5. Conclusions

By using a dataset that covers all macro-regions in Poland in 2018, using the two-stage nested Theil index and conducting regression analysis, our results suggest that mortality rate (as an estimator of a population’s health status) can be understood, in part, as the product of within-country variations in the distribution of inequalities of socioeconomic variables.

These findings provide new evidence in this area, which is a current and developing global topic, and can add supporting arguments in the discussion of the future shape of social and health policy. This study contributes to science in a few ways. We provide new evidence in the area of socioeconomic determinants of health, underlying the importance of the health inequities as a result of unequal distribution of the gas supply and employment in agriculture. We also propose the use of the two-stage nested Theil index for inequity measures of the socioeconomic determinants of health in Poland.

The limitations of the research arise from the range of available data. It would be valuable for Statistics Poland to collect and provide wider and comparable data in this area. The main direction for further research is to focus on policies that foster inequities at all levels (including organisations, communities, powiats, voivodeships, macro-regions and the nation) and elements of the built environment that are critical drivers of inequity. Furthermore, descriptive work should aim to identify priority areas for explanatory and interventional studies.

## Figures and Tables

**Table 1:** Selected existing research on the socioeconomic determinants of health.

Author(s) of Research	Health-Outcome Measures	Examined Socioeconomic Determinants
Zhou, Z., Fang, Y., Zhou, Z., Li, D., Wang, D., Li, Y., Lu, L., Chen, J.G.G. [57].	Health-related quality of life.	Income/urban and rural/educational status.
Barraza-Lloréns, M., Panopoulou, G., Díaz, B.Y. [58].	Self-assessed health, physical limitations, and chronic illness.	Three standard-of-living measures: household income, wealth, and expenditure. Area of residence, geographic region, education, employment, ethnicity, and health insurance.
Chauvel, L., Leist, A.K. [59].	Self-rated health.	Income, education, occupation.
Vásquez, F., Paraje, G., and Estay, M. [60].	Self-assessed health status and physical limitations.	Ethnicity, employment status, health insurance, and region of residence.
Chiu, S.Y.R., Yang, Z. [61].	Health-related quality of life.	Family income, medical insurance coverage.
Wang, R., Liu, Y., Lu, Y., Zhang, J., Liu, P., Yao, Y., Grekousis, G. [62].	Mental health indicators—depression and anxiety, the physical health indicators—the self-rated health condition (SRH) and chronic diseases.	Six perceptual attributes of the built environment: wealth, safety, liveliness, depression, bore and beauty.
Dilmaghani, M. [63].	Self-rated health.	Education, gender.
Basu, A., Jones, A.M., Dias, P.R. [64].	Depression and cigarette smoking.	Schooling systems.
Petrovic, D., de Mestral, C., Bochud, M., Bartley, M., Kivimäki, M., Vineis, P., Mackenbach, J., Stringhini, S. [65].	Cardiometabolic disorders, mortality.	Health behaviours: smoking, alcohol consumption, physical activity and diet.
Øvrum, A., Gustavsen, G.W., Rickertsen, K. [66].	Self-assessed health.	Income, education.
Kabaya, K. [67].	Health spending.	Forest environments.
Hanibuchi, T., Nakaya, T., Honjo, K. [68].	Health-related outcome—self-rated health (SRH), smoking, physical activity.	Income, education, occupation, and subjective social class identification.
Lyu, Y., Forsyth, A., and Worthington, S. [69].	Self-rated health.	Built environment—types of living buildings, having a household smoker, weekly exercise.
Amerio, A., Brambilla, A., Morganti, A., Aguglia, A., Bianchi, D., Santi, F., Costantini, L., Odone, A., Costanza, A., Signorelli, C., Serafini, G., Amore, M., Capolongo, S. [70].	Mental health—depression.	Housing design.
Hone, T., Mirelman, A.J., Rasella, D., Paes-Sousa, R., Barreto, M.L., Rocha, R., Millett, C. [71].	Mortality.	Social protection expenditure.
Alaei, K., Akgüngör, S., Chao, W.-F., Hasan, S., Marshall, A., Schultz, E., Alaei, A. [72].	Mortality rate under 5, mortality rate neonatal, immunisation of diphtheria, pertussis and tetanus (DPT), immunisation of measles, lifetime risk of maternal death (%), life expectancy at birth.	Protection of women’s economic and social rights (WESR): - Physical Integrity Index- Empowerment Rights Index - Women’s Political Rights - Independence of the Judiciary
Ajide, K.B., Alimi, O.Y. [73].	Human life longevity, infant deaths.	Carbon emission.
Krueger, P.M., Rogers, R.G., Hummer, R.A., Leclere, F.B., Bond Huie, S.A. [74].	Mortality rate.	Income, age.
Buttazzoni, A., Doherty, S., and Minaker, L. [75].	Mental health—depression.	Urbanization.
Kim, M., Woo, B., Kim, H.-J., Yi, E., Hong, S. [76].	Perceived stress, depressive symptoms, suicidal ideation, and life satisfaction.	Housing environment—home ownership and perceived house accessibility.
Lei, L., Lin, Z. [77].	Self-rated health.	Neighbourhood types, social cohesion, availability of social institutions in the residents’ committees, water quality.
Tsalta-Mladenov, M., Andonova, S. [78].	Health-related quality of life.	Age, sex, education, working activity.
Wirayuda, A.A.B., Chan, M.F. [79].	Life expectancy.	Infant mortality rate, literacy rate, education level, socioeconomic status, population growth, gender, gross domestic product, income level, unemployment rate, inflation rate, smoking rate, pollution, vaccinations, health care resources, health care facilities, the number of the health care professionals, public health expenditure.
Sleight, A.G., Lyons, K.D., Vigen, C., Macdonald, H., Clark, F. [80].	Health-related quality of life.	Income.
Åström, M., Persson, C., Lindén-Boström, M., Rolfson, O., Burström, K. [81].	Health-related quality of life.	Sex, age, self-reported comorbidity, parents’ occupational status.
Nigusso, F.T., Mavhandu-Mudzusi, A.H. [82].	Health-related quality of life, Global Physical Health, Global Mental Health.	Gender, age, unemployment, household food insecurity and co-morbidities.
Aistov, A., Aleksandrova, E., Gerry, C.J. [83].	Self-assessed health.	Supplemental health insurance and the utilization of health care; health behaviours: visits to doctor, cigarette and alcohol consumption, physical exercise, body mass index; monthly salary, education, chronic disease, if household has children under 3 years, gender, place of living.
Kim, Y., Schneider, T., Faß, E., Lochbaum, M. [84].	Self-rated health.	Education, household income levels.
Dunn, J.R., Walker, J.D., Graham, J., Weiss, C.B. [85].	Self-reported health.	Social support, type of housing, gender.
Miura, K., Takamori, A., Hamazaki, K., Tsuchida, A., Tanaka, T., Origasa, H., Inadera, H. [86].	Health-related quality of life.	Dietary pattern.
Kim, J.-H., Park, E.-Ch. [87].	Health-related quality of life, overall quality of life.	Household income, education levels.

Source: Authors’ study.

**Table 2:** Relations between macro-regions, regions and voivodeships in Poland.

Macro-Region (NUTS 1)	Region (NUTS 2)	Voivodeship
South-West	Lower-Silesian (DL) Opole (O)	Lower-Silesian (DL) Opole (O)
South	Lesser Poland (MŁ) Silesian (ŚL)	Lesser Poland (MŁ) Silesian (ŚL)
North-West	Lubuskie (LU) Wielkopolska (WL) Zachodniopomorskie (ZP)	Lubuskie (LU) Wielkopolska (WL) Zachodniopomorskie (ZP)
North	Kuyavian-Pomeranianranian (K-P) Warmian-Masurian (W-M) Pomeranian (PO)	Kuyavian-Pomeranianranian(K-P) Warmian-Masurian (W-M) Pomeranian (PO)
Central	Łódż (Ł) Świętokrzyskie (ŚW)	Łódż (Ł) Świętokrzyskie (ŚW)
East	Lubelskie (LE) Podkarpackie (PK) Podlaskie (PL)	Lubelskie (LE) Podkarpackie (PK) Podlaskie (PL)
Masovian	Warsaw- capital (W-Stoł) Masovian-regional (MAZ_R)	Masovian (MAZ)

Source: Statistics Poland.

**Table 3:** Socioeconomic determinants of health.

Category	Variable	Description
Economic	IN—Income of powiat	Total income of powiat in PLN per capita
Education	EDE—Gross scholarization ratios for elementary level	The number of pupils enrolled in elementary school to the number of pupils who qualify for elementary education.
	EDJH—Gross scholarization ratios for junior high level	The number of pupils enrolled in junior high school to the number of pupils who qualify for junior high school education.
Employment	EA—Employment rate in agriculture	The percentage of the population aged 15–64 working in agriculture, forestry, hunting and fishing.
	EI—Employment rate in industry	The percentage of the population aged 15–64 working in industry and construction.
	ES—Employment rate in services	The percentage of the population aged 15–64 working in the trades, repairing of vehicles, transport and the warehouse industry, accommodation and catering and information and communication.
	EF—Employment rate in financial sector	The percentage of the population aged 15–64 working in the financial and insurance sector and real estate market.
	UR—Unemployment rate	The number of unemployed people as a percentage of the labour force.
	WAP—Working-age population	The percentage of the working-age population.
Demography	FR—Feminization ratio	Females per 100 males.
	OR—Old-age dependency ratio	Population in the post-production age to 100 people of working age.
Built environment	WS—Water supply	The percentage of people using the water supply system.
	SS—Sewage system	The percentage of people using the sewage system.
	GS—Gas supply	The percentage of people using a gas supply system.
	F—Forest area	Forest area in hectares per capita.
	GR—Green area other than forest	Green area (parks, lawns, etc.) in hectares per capita.
	DIS—Cultural buildings adapted for the disabled	Cultural buildings adapted for the disabled per 1 square km.

Source: Authors’ study.

**Table 4:** Descriptive statistics of socioeconomic determinants of health in Poland.

	Mean	Median	Max.	Min.	Std.dev.	Variance
IN—Income of powiat	4958.0250	4691.6984	9564.0381	3743.9767	837.5420	70,1476.6483
EDE—Gross scholarization ratios for elementary level	0.9409	0.9401	1.1260	0.7605	0.0571	0.0033
EDJH—Gross scholarization ratios for junior high level	0.9916	0.9809	1.4000	0.6032	0.1041	0.0108
EA—Employment rate in agriculture	0.1338	0.1011	0.5023	0.0008	0.1080	0.0117
EI—Employment rate in industry	0.1350	0.1228	0.7757	0.0190	0.0760	0.0058
ES—Employment rate in service sector	0.0782	0.0610	1.2904	0.0153	0.0769	0.0059
EF—Employment rate in financial sector	0.0104	0.0068	0.3084	0.0016	0.0187	0.0003
UR—Unemployment rate	0.0481	0.0439	0.1435	0.0110	0.0230	0.0005
WAP—Working-age population	0.6106	0.6122	0.6444	0.5580	0.0133	0.0002
FR- Feminization ratio	0.5114	0.5096	0.5442	0.4892	0.0091	0.0001
OR—Old-age dependency ratio	0.2084	0.2060	0.3147	0.1386	0.0250	0.0006
WS—Water supply	0.9133	0.9477	1.0000	0.2264	0.1016	0.0103
SS—Sewage supply	0.6446	0.6529	0.9999	0.1274	0.1927	0.0371
GS—Gas supply	0.5028	0.4234	9.7775	0.0000	0.7342	0.5391
F—Forest area	0.3550	0.2606	3.6629	0.0005	0.3968	0.1574
GR—Green area other than forest	0.0033	0.0029	0.0160	0.0002	0.0022	0.0000
DIS—Cultural buildings adapted for the disabled	0.0001	0.0001	0.0005	0.0000	0.0001	0.0000

Source: Authors’ calculations.

**Table 5:** Correlations between independent variables and the mortality rate.

Independent Variable	Spearman’s Rank Correlation Coefficient (Absolute Value)	Significant Correlation?
IN—Income of powiat	0.19	Yes
EDE—Gross scholarization ratios for elementary level	0.05	No
EDJH—Gross scholarization ratios for junior high level	0.06	No
EA—Employment rate in agriculture	0.12	Yes
EI—Employment rate in industry	0.21	Yes
ES—Employment rate in services sector	0.15	Yes
EF—Employment rate in financial sector	0.05	No
UR—Unemployment rate	0.20	Yes
WAP—Working-age population	0.39	Yes
FR—Feminization ratio	0.18	Yes
OR—Old-age dependency ratio	0.65	Yes
WS—Water supply	0.02	No
SS—Sewage supply	0.23	Yes
GS—Gas supply	0.22	Yes
F—Forest area	0.01	No
GR—Green area other than forest	0.04	No
DIS—Cultural buildings adapted for the disabled	0.16	Yes

Source: Authors’ calculations.

**Table 6:** National overall and component inequality of socioeconomic determinants of health distribution.

Variable	*TWP*	*TBP*	*TBR*	*T*
IN—Income of powiat	0.0091	0.0003	0.0019	0.0113
EDE—Gross scholarization ratios for elementary level	0.0009	0.0140	0.0003	0.0151
EDJH—Gross scholarization ratios for junior high level	0.0029	0.0150	0.0003	0.0182
EA—Employment rate in agriculture	0.1466	0.0393	0.0442	0.2300
EI—Employment rate in industry	0.0393	0.0235	0.0047	0.0675
ES—Employment rate in service sector	0.0996	0.0185	0.0118	0.1299
EF—Employment rate in financial sector	0.2439	0.0180	0.0579	0.3198
UR—Unemployment rate	0.0344	0.0238	0.0101	0.0684
WAP—Working-age population	0.0001	0.0000	0.0000	0.0001
FR—Feminization ratio	0.0007	0.0000	0.0000	0.0007
OR—Old-age dependency ratio	0.0033	0.0003	0.0004	0.0040
WS—Water supply	0.0025	0.0003	0.0003	0.0031
SS—Sewage supply	0.0152	0.0012	0.0012	0.0175
GS—Gas supply	0.1491	0.0203	0.0068	0.1762
F—Forest area	0.1754	0.0237	0.0405	0.2396
GR—Green area other than forest	0.4505	0.0062	0.0077	0.4644
DIS—Cultural buildings adapted for the disabled	0.1087	0.0063	0.0081	0.1230

Source: Authors’ calculations.

**Table 7:** The HHI values for the socioeconomic determinants of health in Poland in 2018.

Variable/Macro-Region	South-West	South	North-West	North	Central	East	Masovian
IN—Income of powiat	1536	787	876	967	1604	997	2633
EDE—Gross scholarization ratios for elementary level	1480	754	894	890	1508	985	1668
EDJH—Gross scholarization ratios for junior high level	1465	751	884	880	1517	977	1647
EA—Employment rate in agriculture	1725	1276	1282	903	1951	1027	1439
EI—Employment rate in industry	1540	803	984	906	1600	1115	1782
ES—Employment rate in service sector	2059	1022	986	1408	1957	1153	3952
EF—Employment rate in financial sector	3365	1667	1458	1810	3176	1378	7518
UR—Unemployment rate	1514	756	965	875	1646	985	3059
WAP—Working-age population	1469	750	874	887	1526	978	3252
FR—Feminization ratio	1476	755	873	902	1538	983	3282
OR—Old-age dependency ratio	1491	774	866	948	1578	986	1924
WS—Water supply	1471	785	874	902	1541	1002	3289
SS—Sewage supply	1503	801	863	967	1749	1030	3484
GS—Gas supply	1579	817	938	1205	2493	1303	3637
F—Forest area	1901	1033	1213	1074	2055	1027	1559
GR—Green area other than forest	1637	974	903	1055	1927	1208	2831
DIS—Cultural buildings adapted for the disabled	1635	852	1028	901	1664	1010	1454

Source: Authors’ calculations.

**Table 8:** Parameters of the regression models.

Independent Variable	Preliminary Model		Final Model	
	a	*p*-Value	a	*p*-Value
IN—Income of powiat	0.0002	0.482		
EA—Employment rate in agriculture	−0.0117	0.194	−0.0215	<0.001
EI—Employment rate in industry	0.0057	0.561		
ES—Employment rate in service sector	0.0057	0.685		
UR—Unemployment rate	0.0511	0.027	0.0427	0.049
WAP—Working-age population	0.1704	0.003	0.2173	<0.001
FR—Feminization ratio	−0.0653	0.026	−0.0940	<0.001
OR—Old-age dependency ratio	0.3536	< 0.001	0.3567	<0.001
SS—Sewage supply	−0.0285	< 0.001	−0.0356	<0.001
GS—Gas supply	−0.0120	< 0.001	−0.0119	<0.001
DIS—Cultural buildings adapted for the disabled	−364.3296	0.553		
Intercept	−2.6892		−1.5577	
R square	0.7906		0.7220	

Source: Authors’ calculations.

## Data Availability

The data presented in this study are available on request from the corresponding author.

## Supplementary Materials

The following are available online at https://www.mdpi.com/article/10.3390/ijerph182010856/s1, Territorial structure.
