# Supplementary material for: Socioeconomic Determinants of Health and Their Unequal Distribution in Poland
Source: Int J Environ Res Public Health. 2021 Oct 15;18(20):10856. doi: 10.3390/ijerph182010856 (PMC8536126; doi:10.3390/ijerph182010856)
Supplement: Supplementary file 1 [file ijerph-18-10856-s001.zip › ijerph-1354982-supplementary.pdf]

Supplementary material – data			
**Territorial structure**			
**Macro-region**	**Voivodeship**	**Code**	**Powiat**
South-west	Lower-Silesian	0201000	Powiat bolesławiecki
		0202000	Powiat dzierżoniowski
		0203000	Powiat głogowski
		0204000	Powiat górowski
		0205000	Powiat jaworski
		0206000	Powiat jeleniogórski
		0207000	Powiat kamiennogórski
		0208000	Powiat kłodzki
		0209000	Powiat legnicki
		0210000	Powiat lubański
		0211000	Powiat lubiński
		0212000	Powiat lwówecki
		0213000	Powiat milicki
		0214000	Powiat oleśnicki
		0215000	Powiat oławski
		0216000	Powiat polkowicki
		0217000	Powiat strzeliński
		0218000	Powiat średzki
		0219000	Powiat świdnicki
		0220000	Powiat trzebnicki
		0221000	Powiat wałbrzyski
		0222000	Powiat wołowski
		0223000	Powiat wrocławski
		0224000	Powiat ząbkowicki
		0225000	Powiat zgorzelecki
		0226000	Powiat złotoryjski
		0261000	Powiat m.Jelenia Góra
		0262000	Powiat m.Legnica
		0264000	Powiat m.Wrocław
		0265000	Powiat m.Wałbrzych
North	Kuyavian-Pomeranianranian	0401000	Powiat aleksandrowski
		0402000	Powiat brodnicki
		0403000	Powiat bydgoski
		0404000	Powiat chełmiński
		0405000	Powiat golubsko-dobrzyński
		0406000	Powiat grudziądzki
		0407000	Powiat inowrocławski
		0408000	Powiat lipnowski
		0409000	Powiat mogileński
		0410000	Powiat nakielski
		0411000	Powiat radziejowski
		0412000	Powiat rypiński
		0413000	Powiat sępoleński
		0414000	Powiat świecki
		0415000	Powiat toruński
		0416000	Powiat tucholski
		0417000	Powiat wąbrzeski
		0418000	Powiat włocławski

		0419000	Powiat żniński
		0461000	Powiat m.Bydgoszcz
		0462000	Powiat m.Grudziądz
		0463000	Powiat m.Toruń
		0464000	Powiat m.Włocławek
East	Lubelskie	0601000	Powiat bialski
		0602000	Powiat biłgorajski
		0603000	Powiat chełmski
		0604000	Powiat hrubieszowski
		0605000	Powiat janowski
		0606000	Powiat krasnostawski
		0607000	Powiat kraśnicki
		0608000	Powiat lubartowski
		0609000	Powiat lubelski
		0610000	Powiat łęczyński
		0611000	Powiat łukowski
		0612000	Powiat opolski
		0613000	Powiat parczewski
		0614000	Powiat puławski
		0615000	Powiat radzyński
		0616000	Powiat rycki
		0617000	Powiat świdnicki
		0618000	Powiat tomaszowski
		0619000	Powiat włodawski
		0620000	Powiat zamojski
		0661000	Powiat m.Biała Podlaska
		0662000	Powiat m.Chełm
		0663000	Powiat m.Lublin
		0664000	Powiat m.Zamość
North-West	Lubuskie	0801000	Powiat gorzowski
		0802000	Powiat krośnieński
		0803000	Powiat międzyrzecki
		0804000	Powiat nowosolski
		0805000	Powiat słubicki
		0806000	Powiat strzelecko-drezdenecki
		0807000	Powiat sulęciński
		0808000	Powiat świebodziński
		0809000	Powiat zielonogórski
		0810000	Powiat żagański
		0811000	Powiat żarski
		0812000	Powiat wschowski
		0861000	Powiat m.Gorzów Wielkopolski
		0862000	Powiat m.Zielona Góra
		1001000	Powiat bełchatowski
		1002000	Powiat kutnowski
		1003000	Powiat łaski
		1004000	Powiat łęczycki
		1005000	Powiat łowicki
		1006000	Powiat łódzki wschodni
		1007000	Powiat opoczyński
		1008000	Powiat pabianicki

**Central**	Łódż	1009000	Powiat pajęczański
		1010000	Powiat piotrkowski
		1011000	Powiat poddębicki
		1012000	Powiat radomszczański
		1013000	Powiat rawski
		1014000	Powiat sieradzki
		1015000	Powiat skierniewicki
		1016000	Powiat tomaszowski
		1017000	Powiat wieluński
		1018000	Powiat wieruszowski
		1019000	Powiat zduńskowolski
		1020000	Powiat zgierski
		1021000	Powiat brzeziński
		1061000	Powiat m.Łódź
		1062000	Powiat m.Piotrków Trybunalski
		1063000	Powiat m.Skierniewice
**South**	Lesser Poland	1201000	Powiat bocheński
		1202000	Powiat brzeski
		1203000	Powiat chrzanowski
		1204000	Powiat dąbrowski
		1205000	Powiat gorlicki
		1206000	Powiat krakowski
		1207000	Powiat limanowski
		1208000	Powiat miechowski
		1209000	Powiat myślenicki
		1210000	Powiat nowosądecki
		1211000	Powiat nowotarski
		1212000	Powiat olkuski
		1213000	Powiat oświęcimski
		1214000	Powiat proszowicki
		1215000	Powiat suski
		1216000	Powiat tarnowski
		1217000	Powiat tatrzański
		1218000	Powiat wadowicki
		1219000	Powiat wielicki
		1261000	Powiat m.Kraków
		1262000	Powiat m.Nowy Sącz
		1263000	Powiat m.Tarnów
		1401000	Powiat białobrzeski
		1402000	Powiat ciechanowski
		1403000	Powiat garwoliński
		1404000	Powiat gostyniński
		1405000	Powiat grodziski
		1406000	Powiat grójecki
		1407000	Powiat kozienicki
		1408000	Powiat legionowski
		1409000	Powiat lipski
		1410000	Powiat łosicki
		1411000	Powiat makowski
		1412000	Powiat miński
		1413000	Powiat mławski

**Masovian**	Masovian	1414000	Powiat nowodworski
		1415000	Powiat ostrołęcki
		1416000	Powiat ostrowski
		1417000	Powiat otwocki
		1418000	Powiat piaseczyński
		1419000	Powiat płocki
		1420000	Powiat płoński
		1421000	Powiat pruszkowski
		1422000	Powiat przasnyski
		1423000	Powiat przysuski
		1424000	Powiat pułtuski
		1425000	Powiat radomski
		1426000	Powiat siedlecki
		1427000	Powiat sierpecki
		1428000	Powiat sochaczewski
		1429000	Powiat sokołowski
		1430000	Powiat szydłowiecki
		1432000	Powiat warszawski zachodni
		1433000	Powiat węgrowski
		1434000	Powiat wołomiński
		1435000	Powiat wyszkowski
		1436000	Powiat zwoleński
		1437000	Powiat żuromiński
		1438000	Powiat żyrardowski
		1461000	Powiat m.Ostrołęka
		1462000	Powiat m.Płock
		1463000	Powiat m.Radom
		1464000	Powiat m.Siedlce
		1465000	Powiat m. st. Warszawa
**South-west**	Opole	1601000	Powiat brzeski
		1602000	Powiat głubczycki
		1603000	Powiat kędzierzyńsko-kozielski
		1604000	Powiat kluczborski
		1605000	Powiat krapkowicki
		1606000	Powiat namysłowski
		1607000	Powiat nyski
		1608000	Powiat oleski
		1609000	Powiat opolski
		1610000	Powiat prudnicki
		1611000	Powiat strzelecki
		1661000	Powiat m.Opole
		1801000	Powiat bieszczadzki
		1802000	Powiat brzozowski
		1803000	Powiat dębicki
		1804000	Powiat jarosławski
		1805000	Powiat jasielski
		1806000	Powiat kolbuszowski
		1807000	Powiat krośnieński
		1808000	Powiat leżajski
		1809000	Powiat lubaczowski
		1810000	Powiat łańcucki

East	Podkarpackie	1811000	Powiat mielecki
		1812000	Powiat niżański
		1813000	Powiat przemyski
		1814000	Powiat przeworski
		1815000	Powiat ropczycko-sędziszowski
		1816000	Powiat rzeszowski
		1817000	Powiat sanocki
		1818000	Powiat stalowowolski
		1819000	Powiat strzyżowski
		1820000	Powiat tarnobrzeski
		1821000	Powiat leski
		1861000	Powiat m.Krosno
		1862000	Powiat m.Przemyśl
		1863000	Powiat m.Rzeszów
		1864000	Powiat m.Tarnobrzeg
East	Podlaskie	2001000	Powiat augustowski
		2002000	Powiat białostocki
		2003000	Powiat bielski
		2004000	Powiat grajewski
		2005000	Powiat hajnowski
		2006000	Powiat kolneński
		2007000	Powiat łomżyński
		2008000	Powiat moniecki
		2009000	Powiat sejneński
		2010000	Powiat siemiatycki
		2011000	Powiat sokólski
		2012000	Powiat suwalski
		2013000	Powiat wysokomazowiecki
		2014000	Powiat zambrowski
		2061000	Powiat m.Białystok
		2062000	Powiat m.Łomża
		2063000	Powiat m.Suwałki
North	Pomeranian	2201000	Powiat bytowski
		2202000	Powiat chojnicki
		2203000	Powiat człuchowski
		2204000	Powiat gdański
		2205000	Powiat kartuski
		2206000	Powiat kościerski
		2207000	Powiat kwidzyński
		2208000	Powiat lęborski
		2209000	Powiat malborski
		2210000	Powiat nowodworski
		2211000	Powiat pucki
		2212000	Powiat słupski
		2213000	Powiat starogardzki
		2214000	Powiat tczewski
		2215000	Powiat wejherowski
		2216000	Powiat sztumski
		2261000	Powiat m.Gdańsk
		2262000	Powiat m.Gdynia
		2263000	Powiat m.Słupsk

		2264000	Powiat m.Sopot
**South**	Silesian	2401000	Powiat będziński
		2402000	Powiat bielski
		2403000	Powiat cieszyński
		2404000	Powiat częstochowski
		2405000	Powiat gliwicki
		2406000	Powiat kłobucki
		2407000	Powiat lubliniecki
		2408000	Powiat mikołowski
		2409000	Powiat myszkowski
		2410000	Powiat pszczyński
		2411000	Powiat raciborski
		2412000	Powiat rybnicki
		2413000	Powiat tarnogórski
		2414000	Powiat bieruńsko-lędziński
		2415000	Powiat wodzisławski
		2416000	Powiat zawierciański
		2417000	Powiat żywiecki
		2461000	Powiat m.Bielsko-Biała
		2462000	Powiat m.Bytom
		2463000	Powiat m.Chorzów
		2464000	Powiat m.Częstochowa
		2465000	Powiat m.Dąbrowa Górnicza
		2466000	Powiat m.Gliwice
		2467000	Powiat m.Jastrzębie-Zdrój
		2468000	Powiat m.Jaworzno
		2469000	Powiat m.Katowice
		2470000	Powiat m.Mysłowice
		2471000	Powiat m.Piekary Śląskie
		2472000	Powiat m.Ruda Śląska
		2473000	Powiat m.Rybnik
		2474000	Powiat m.Siemianowice Śląskie
		2475000	Powiat m.Sosnowiec
		2476000	Powiat m.Świętochłowice
		2477000	Powiat m.Tychy
		2478000	Powiat m.Zabrze
		2479000	Powiat m.Żory
**Central**	Świętokrzyskie	2601000	Powiat buski
		2602000	Powiat jędrzejowski
		2603000	Powiat kazimierski
		2604000	Powiat kielecki
		2605000	Powiat konecki
		2606000	Powiat opatowski
		2607000	Powiat ostrowiecki
		2608000	Powiat pińczowski
		2609000	Powiat sandomierski
		2610000	Powiat skarżyski
		2611000	Powiat starachowicki
		2612000	Powiat staszowski
		2613000	Powiat włoszczowski
		2661000	Powiat m.Kielce

North	WarmianMasurian	2801000	Powiat bartoszycki
		2802000	Powiat braniewski
		2803000	Powiat działdowski
		2804000	Powiat elbląski
		2805000	Powiat ełcki
		2806000	Powiat giżycki
		2807000	Powiat iławski
		2808000	Powiat kętrzyński
		2809000	Powiat lidzbarski
		2810000	Powiat mrągowski
		2811000	Powiat nidzicki
		2812000	Powiat nowomiejski
		2813000	Powiat olecki
		2814000	Powiat olsztyński
		2815000	Powiat ostródzki
		2816000	Powiat piski
		2817000	Powiat szczycieński
		2818000	Powiat gołdapski
		2819000	Powiat węgorzewski
		2861000	Powiat m.Elbląg
		2862000	Powiat m.Olsztyn
North - west	Wielkopolska	3001000	Powiat chodzieski
		3002000	Powiat czarnkowsko-trzcianecki
		3003000	Powiat gnieźnieński
		3004000	Powiat gostyński
		3005000	Powiat grodziski
		3006000	Powiat jarociński
		3007000	Powiat kaliski
		3008000	Powiat kępiński
		3009000	Powiat kolski
		3010000	Powiat koniński
		3011000	Powiat kościański
		3012000	Powiat krotoszyński
		3013000	Powiat leszczyński
		3014000	Powiat międzychodzki
		3015000	Powiat nowotomyski
		3016000	Powiat obornicki
		3017000	Powiat ostrowski
		3018000	Powiat ostrzeszowski
		3019000	Powiat pilski
		3020000	Powiat pleszewski
		3021000	Powiat poznański
		3022000	Powiat rawicki
		3023000	Powiat słupecki
		3024000	Powiat szamotulski
		3025000	Powiat średzki
		3026000	Powiat śremski
		3027000	Powiat turecki
		3028000	Powiat wągrowiecki
		3029000	Powiat wolsztyński
		3030000	Powiat wrzesiński

		3031000	Powiat złotowski
		3061000	Powiat m.Kalisz
		3062000	Powiat m.Konin
		3063000	Powiat m.Leszno
		3064000	Powiat m.Poznań
**North-West**	Zachodniopomorskie	3201000	Powiat białogardzki
		3202000	Powiat choszczeński
		3203000	Powiat drawski
		3204000	Powiat goleniowski
		3205000	Powiat gryficki
		3206000	Powiat gryfiński
		3207000	Powiat kamieński
		3208000	Powiat kołobrzeski
		3209000	Powiat koszaliński
		3210000	Powiat myśliborski
		3211000	Powiat policki
		3212000	Powiat pyrzycki
		3213000	Powiat sławieński
		3214000	Powiat stargardzki
		3215000	Powiat szczecinecki
		3216000	Powiat świdwiński
		3217000	Powiat wałecki
		3218000	Powiat łobeski
		3261000	Powiat m.Koszalin
		3262000	Powiat m.Szczecin
		3263000	Powiat m.Świnoujście

Code	Powiat	Total population	Total income - powiat in PLN	Gross scholarization ratios		Elementary school	Junior high level school
				Elementary school	Junior high level school		
				in %	in %	in persons	in persons
0201000	Powiat bolesławiecki	90 200	430 027 376,59	94,86	96,01	5 469	2 459
0202000	Powiat dzierżoniowski	101 437	421 986 800,94	90,69	105,34	5 143	2 647
0203000	Powiat głogowski	89 541	464 308 303,99	96,07	97,85	5 885	2 521
0204000	Powiat górowski	35 182	168 664 355,18	89,61	93,30	2 060	1 022
0205000	Powiat jaworski	50 545	243 401 452,82	87,81	93,87	2 695	1 282
0206000	Powiat jeleniogórski	63 855	326 791 321,08	79,73	76,26	2 915	1 338
0207000	Powiat kamiennogórski	43 621	186 916 636,48	88,89	92,93	2 311	1 151
0208000	Powiat kłodzki	159 337	729 907 981,91	91,01	93,05	8 254	3 773
0209000	Powiat legnicki	55 326	255 320 569,07	83,92	78,05	3 015	1 229
0210000	Powiat lubański	54 699	265 601 783,02	91,75	94,44	2 942	1 444
0211000	Powiat lubiński	106 254	593 679 752,70	98,38	108,96	6 558	3 210
0212000	Powiat lwówecki	46 059	214 592 016,43	86,41	93,09	2 277	1 159
0213000	Powiat milicki	37 093	183 372 313,85	94,41	95,61	2 451	1 135
0214000	Powiat oleśnicki	107 062	466 945 610,15	96,24	101,78	6 865	3 150
0215000	Powiat oławski	76 595	367 961 655,78	96,72	98,13	5 106	2 083
0216000	Powiat polkowicki	62 982	433 155 474,25	92,43	85,65	4 046	1 635
0217000	Powiat strzeliński	43 773	203 204 447,17	91,89	97,44	2 463	1 184
0218000	Powiat średzki	54 269	269 734 583,92	90,89	80,79	3 471	1 262
0219000	Powiat świdnicki	157 615	749 166 321,90	94,93	98,38	9 009	4 038
0220000	Powiat trzebnicki	84 886	419 491 842,59	93,05	93,14	5 554	2 395
0221000	Powiat wałbrzyski	56 004	258 217 021,89	86,91	84,97	2 815	1 209
0222000	Powiat wołowski	47 056	209 183 604,04	96,89	105,10	2 695	1 308
0223000	Powiat wrocławski	146 060	864 433 370,57	95,99	78,96	11 478	3 479
0224000	Powiat ząbkowicki	65 428	283 482 206,50	87,53	96,26	3 354	1 583
0225000	Powiat zgorzelecki	90 003	443 798 045,44	94,30	95,37	5 018	2 298
0226000	Powiat złotoryjski	43 869	203 170 877,63	89,13	88,54	2 503	993
0261000	Powiat m.Jelenia Góra	79 480	449 494 533,81	110,11	128,90	4 521	2 303
0262000	Powiat m.Legnica	99 752	547 462 727,20	100,47	109,87	5 827	2 716
0264000	Powiat m.Wrocław	640 648	4 456 648 391,83	100,21	113,74	36 093	14 950
0265000	Powiat m.Wałbrzych od 201	112 594	621 260 396,03	96,37	91,07	5 728	2 473
0401000	Powiat aleksandrowski	55 274	251 073 050,38	93,20	99,25	3 240	1 621

0402000	Powiat brodnicki	78 898	367 735 830,62	94,73	100,60	5 486	2 460
0403000	Powiat bydgoski	117 325	600 646 492,46	89,06	80,96	8 034	3 238
0404000	Powiat chełmiński	52 193	231 183 566,52	89,60	89,94	3 232	1 502
0405000	Powiat golubsko-dobrzyński	45 140	203 729 993,82	93,47	97,57	2 858	1 426
0406000	Powiat grudziądzki	40 271	204 455 460,87	83,88	90,68	2 584	1 230
0407000	Powiat inowrocławski	160 647	719 344 685,00	94,08	98,40	9 156	4 278
0408000	Powiat lipnowski	66 062	324 106 151,67	92,01	101,63	4 313	2 129
0409000	Powiat mogileński	45 821	198 442 946,73	93,83	107,67	2 745	1 417
0410000	Powiat nakielski	86 590	401 766 491,40	90,39	98,49	5 592	2 759
0411000	Powiat radziejowski	40 714	188 114 024,41	94,69	104,58	2 325	1 214
0412000	Powiat rypiński	43 826	206 737 498,25	93,22	98,31	2 853	1 383
0413000	Powiat sępoleński	41 147	193 062 660,82	88,80	93,19	2 718	1 181
0414000	Powiat świecki	99 281	490 344 056,63	93,78	95,78	6 462	2 945
0415000	Powiat toruński	106 935	501 672 075,34	85,26	80,91	7 181	3 109
0416000	Powiat tucholski	48 374	252 912 779,45	93,40	97,42	3 225	1 465
0417000	Powiat wąbrzeski	34 413	159 300 529,00	91,05	98,09	2 101	1 010
0418000	Powiat włocławski	86 352	410 318 905,50	86,82	87,54	4 777	2 250
0419000	Powiat żniński	70 413	325 796 571,09	92,87	95,97	4 388	2 042
0461000	Powiat m.Bydgoszcz	350 178	2 109 052 562,28	98,37	109,66	19 375	9 020
0462000	Powiat m.Grudziądz	95 045	571 484 911,76	98,87	109,31	5 974	2 772
0463000	Powiat m.Toruń	202 074	1 196 973 015,11	102,92	117,23	11 961	5 769
0464000	Powiat m.Włocławek	110 802	657 445 132,08	96,90	116,57	6 016	3 316
0601000	Powiat bialski	111 391	547 290 387,01	87,97	92,47	6 519	3 246
0602000	Powiat biłgorajski	101 435	463 081 946,90	92,57	96,04	5 884	2 985
0603000	Powiat chełmski	78 228	344 787 840,90	82,77	86,62	4 277	1 984
0604000	Powiat hrubieszowski	63 698	254 575 960,21	87,57	95,11	3 230	1 673
0605000	Powiat janowski	46 057	211 517 959,70	91,72	90,42	2 564	1 312
0606000	Powiat krasnostawski	63 925	249 661 417,34	90,96	98,95	3 154	1 742
0607000	Powiat kraśnicki	96 043	405 777 491,55	91,39	95,26	5 230	2 615
0608000	Powiat lubartowski	88 789	406 148 951,11	92,23	97,47	5 537	2 710
0609000	Powiat lubelski	154 387	713 888 498,87	78,97	73,91	8 769	3 686
0610000	Powiat łęczyński	57 457	264 183 523,67	93,10	98,31	3 542	1 756
0611000	Powiat łukowski	107 449	499 087 031,27	96,11	102,09	7 355	3 640
0612000	Powiat opolski	59 726	253 173 636,32	92,41	98,64	3 493	1 724
0613000	Powiat parczewski	34 954	166 857 150,87	90,41	89,88	1 937	906
0614000	Powiat puławski	113 762	545 472 577,89	96,89	102,28	6 431	3 292
0615000	Powiat radzyński	59 278	282 272 141,81	95,72	100,74	3 841	2 008

0616000	Powiat rycki	56 166	247 800 456,60	95,10	106,14	3 264	1 701
0617000	Powiat świdnicki	72 039	293 363 789,43	91,32	92,93	4 059	1 877
0618000	Powiat tomaszowski	83 715	359 942 753,23	91,13	97,53	4 545	2 341
0619000	Powiat włodawski	38 668	179 998 556,96	90,67	101,39	2 200	1 101
0620000	Powiat zamojski	106 935	448 346 231,45	82,88	79,44	5 437	2 556
0661000	Powiat m.Biała Podlaska	57 352	322 087 982,89	110,57	125,80	3 967	1 848
0662000	Powiat m.Chełm	62 670	342 044 694,39	103,91	127,52	3 624	1 952
0663000	Powiat m.Lublin	339 682	2 241 907 783,26	106,31	114,12	21 073	9 601
0664000	Powiat m.Zamość	63 813	400 722 908,43	111,04	131,38	4 010	2 165
0801000	Powiat gorzowski	71 549	350 787 359,84	86,09	83,19	4 503	1 967
0802000	Powiat krośnieński	55 245	242 878 845,25	91,87	91,22	3 244	1 378
0803000	Powiat międzyrzecki	58 024	273 201 476,05	92,94	98,20	3 538	1 566
0804000	Powiat nowosolski	86 634	393 000 315,70	90,41	92,79	5 338	2 318
0805000	Powiat słubicki	47 101	228 684 789,91	96,39	96,37	3 144	1 416
0806000	Powiat strzelecko-drezdenec	49 366	230 880 368,70	91,96	93,18	3 107	1 368
0807000	Powiat sulęciński	35 297	168 665 856,62	91,71	88,04	2 299	893
0808000	Powiat świebodziński	55 840	258 736 155,82	95,65	100,40	3 507	1 601
0809000	Powiat zielonogórski	75 750	354 169 014,75	87,64	94,60	4 703	2 122
0810000	Powiat żagański	79 583	356 595 632,71	88,59	88,02	4 469	1 909
0811000	Powiat żarski	96 876	428 479 050,56	94,64	95,83	5 565	2 660
0812000	Powiat wschowski	39 065	172 925 360,37	94,17	100,00	2 547	1 166
0861000	Powiat m.Gorzów Wielkopo	123 921	756 278 502,58	100,46	116,16	7 787	3 682
0862000	Powiat m.Zielona Góra	140 297	923 401 546,59	103,35	108,10	9 092	3 931
1001000	Powiat bełchatowski	112 997	758 283 611,11	94,82	105,28	7 253	3 225
1002000	Powiat kutnowski	97 295	413 758 838,27	97,53	104,92	5 321	2 554
1003000	Powiat łaski	50 103	211 508 226,41	95,20	105,64	2 846	1 428
1004000	Powiat łęczycki	50 143	234 873 868,10	94,06	106,87	2 844	1 397
1005000	Powiat łowicki	78 616	333 401 684,80	97,35	102,98	4 762	2 255
1006000	Powiat łódzki wschodni	71 705	338 326 836,99	88,48	95,88	4 390	2 112
1007000	Powiat opoczyński	76 623	341 223 351,23	95,10	99,02	5 006	2 386
1008000	Powiat pabianicki	119 289	504 446 053,78	92,97	102,29	6 657	3 091
1009000	Powiat pajęczański	51 597	286 671 440,20	96,00	97,81	3 000	1 420
1010000	Powiat piotrkowski	91 315	425 557 528,84	92,38	94,59	5 825	2 749
1011000	Powiat poddębicki	41 205	205 948 649,06	93,43	96,21	2 350	1 069
1012000	Powiat radomszczański	113 315	490 323 653,20	95,18	99,36	6 559	3 078
1013000	Powiat rawski	48 808	225 548 654,75	96,43	107,07	3 109	1 541
1014000	Powiat sieradzki	118 240	516 343 811,98	95,45	98,80	7 070	3 327

1015000	Powiat skierniewicki	38 195	177 205 980,06	89,34	81,63	2 396	962
1016000	Powiat tomaszowski	117 259	562 582 220,33	95,68	99,90	7 285	3 362
1017000	Powiat wieluński	76 699	336 758 321,84	96,24	100,94	4 699	2 260
1018000	Powiat wieruszowski	42 213	191 321 696,54	96,00	103,73	2 707	1 331
1019000	Powiat zduńskowolski	66 766	270 697 756,11	96,54	103,17	4 145	1 986
1020000	Powiat zgierski	165 916	747 172 951,71	93,66	94,01	9 890	4 255
1021000	Powiat brzeziński	30 890	139 197 983,72	88,48	93,71	1 743	814
1061000	Powiat m.Łódź	685 285	4 076 467 290,05	99,35	112,65	35 636	17 363
1062000	Powiat m.Piotrków Trybuna	73 670	432 733 130,18	96,15	107,55	4 453	2 220
1063000	Powiat m.Skierniewice	48 178	269 847 808,66	106,23	117,11	3 389	1 503
1201000	Powiat bocheński	106 514	494 871 505,20	95,38	97,55	7 465	3 305
1202000	Powiat brzeski	93 206	399 857 432,69	92,36	98,46	6 030	2 893
1203000	Powiat chrzanowski	125 385	510 820 403,92	95,56	100,19	6 955	3 187
1204000	Powiat dąbrowski	59 350	232 571 930,76	88,65	92,21	3 192	1 640
1205000	Powiat gorlicki	109 104	507 988 118,45	94,72	97,39	7 229	3 316
1206000	Powiat krakowski	277 145	1 269 943 810,42	90,43	86,05	17 854	7 423
1207000	Powiat limanowski	131 523	653 261 209,55	94,91	98,21	9 991	4 764
1208000	Powiat miechowski	49 137	202 494 281,50	95,88	95,31	2 833	1 309
1209000	Powiat myślenicki	127 028	575 862 046,78	96,71	102,42	9 245	4 343
1210000	Powiat nowosądecki	216 176	1 113 147 050,21	91,60	92,47	15 572	7 240
1211000	Powiat nowotarski	191 508	861 880 929,98	94,25	96,99	12 903	6 139
1212000	Powiat olkuski	112 035	478 999 088,48	94,38	100,91	6 361	2 952
1213000	Powiat oświęcimski	153 737	691 432 534,16	96,16	102,78	9 325	4 279
1214000	Powiat proszowicki	43 523	193 910 365,02	96,14	98,58	2 607	1 244
1215000	Powiat suski	84 255	384 885 277,11	94,02	97,16	5 634	2 615
1216000	Powiat tarnowski	201 570	835 827 643,99	87,76	84,87	11 935	5 423
1217000	Powiat tatrzański	68 146	341 042 998,21	90,83	91,55	4 076	1 897
1218000	Powiat wadowicki	160 130	698 799 770,97	96,48	98,51	10 755	4 937
1219000	Powiat wielicki	127 078	626 139 653,24	95,60	97,93	9 337	3 933
1261000	Powiat m.Kraków	771 069	5 307 143 311,36	101,49	115,59	43 505	20 041
1262000	Powiat m.Nowy Sącz	83 896	634 875 814,85	102,49	112,39	5 551	2 803
1263000	Powiat m.Tarnów	109 062	680 624 207,93	108,71	129,23	6 364	3 397
1401000	Powiat białobrzeski	33 551	160 161 278,29	94,43	95,26	2 233	1 068
1402000	Powiat ciechanowski	89 670	427 172 484,78	96,61	103,70	5 393	2 584
1403000	Powiat garwoliński	108 993	492 182 418,49	97,00	105,54	7 572	3 851
1404000	Powiat gostyniński	45 203	175 775 563,59	91,40	93,84	2 511	1 185
1405000	Powiat grodziski	94 091	584 724 352,04	105,08	104,61	7 744	3 122

1406000	Powiat grójecki	98 451	426 949 889,15	99,44	101,83	6 498	3 150
1407000	Powiat kozienicki	60 472	306 165 306,45	94,35	100,17	3 472	1 696
1408000	Powiat legionowski	116 775	618 860 555,58	99,19	100,67	9 433	3 838
1409000	Powiat lipski	34 214	142 427 444,72	92,61	108,12	1 839	1 009
1410000	Powiat łosicki	31 023	141 536 137,38	92,52	109,30	1 855	955
1411000	Powiat makowski	45 247	226 517 907,16	93,39	104,71	2 814	1 465
1412000	Powiat miński	153 824	743 263 735,29	103,65	110,95	11 808	5 222
1413000	Powiat mławski	73 102	338 836 360,22	96,03	98,83	4 702	2 123
1414000	Powiat nowodworski	79 291	422 431 535,53	98,88	95,27	5 343	2 250
1415000	Powiat ostrołęcki	88 735	420 281 292,31	87,00	87,43	5 578	2 661
1416000	Powiat ostrowski	72 782	325 657 529,31	95,82	100,97	4 409	2 212
1417000	Powiat otwocki	124 036	611 854 050,95	97,87	99,32	8 740	3 806
1418000	Powiat piaseczyński	184 951	1 101 473 230,84	102,01	106,66	15 567	6 887
1419000	Powiat płocki	111 088	523 664 792,49	85,37	91,16	6 432	3 194
1420000	Powiat płoński	87 409	410 377 589,35	97,79	103,43	5 611	2 679
1421000	Powiat pruszkowski	164 240	954 722 424,36	99,77	96,30	11 830	4 691
1422000	Powiat przasnyski	52 824	255 732 097,67	93,32	96,98	3 444	1 658
1423000	Powiat przysuski	41 922	187 358 520,20	96,16	113,35	2 354	1 420
1424000	Powiat pułtuski	51 809	241 212 850,13	97,78	98,40	3 346	1 557
1425000	Powiat radomski	152 117	673 703 584,06	89,59	87,62	9 678	4 433
1426000	Powiat siedlecki	81 352	368 576 700,18	89,43	100,12	5 030	2 601
1427000	Powiat sierpecki	52 196	241 540 038,56	94,72	95,04	3 113	1 476
1428000	Powiat sochaczewski	85 105	411 129 002,11	100,49	109,27	5 705	2 719
1429000	Powiat sokołowski	54 217	237 429 366,06	95,15	93,23	3 112	1 379
1430000	Powiat szydłowiecki	39 864	175 189 081,03	89,22	93,50	2 283	1 137
1432000	Powiat warszawski zachodni	117 007	752 708 133,60	95,88	92,07	8 619	3 526
1433000	Powiat węgrowski	66 174	320 960 764,89	96,70	109,87	4 091	2 118
1434000	Powiat wołomiński	245 357	1 202 568 439,77	100,77	102,81	19 739	8 425
1435000	Powiat wyszkowski	74 065	350 498 326,07	97,06	102,02	5 102	2 374
1436000	Powiat zwoleński	36 328	169 118 896,38	93,17	96,69	2 199	1 056
1437000	Powiat żuromiński	38 944	172 307 261,30	93,53	102,70	2 365	1 179
1438000	Powiat żyrardowski	75 848	421 909 185,65	96,61	96,42	5 073	2 144
1461000	Powiat m.Ostrołęka	52 262	359 015 242,86	112,60	129,16	3 607	1 750
1462000	Powiat m.Płock	120 000	1 080 064 586,04	108,60	117,24	7 866	3 631
1463000	Powiat m.Radom	213 029	1 203 401 892,29	100,69	112,18	13 069	6 504
1464000	Powiat m.Siedlce	77 872	506 910 412,15	106,74	110,70	5 658	2 462
1465000	Powiat m. st. Warszawa	1 777 972	17 004 591 865,17	101,73	122,63	113 392	52 010

1601000	Powiat brzeski	90 275	401 753 809,32	91,03	92,57	5 343	2 225
1602000	Powiat głubczycki	45 883	195 699 008,60	88,30	93,37	2 291	1 157
1603000	Powiat kędzierzyńsko-kozie	94 487	415 288 958,68	93,58	92,52	4 718	2 133
1604000	Powiat kluczborski	65 837	312 383 556,48	93,11	101,69	3 304	1 683
1605000	Powiat krapkowicki	63 942	261 371 065,20	94,68	95,23	3 170	1 521
1606000	Powiat namysłowski	42 688	181 211 447,96	93,74	105,60	2 553	1 211
1607000	Powiat nyski	136 946	557 743 978,40	90,56	95,79	6 892	3 351
1608000	Powiat oleski	64 602	254 796 822,50	96,16	99,24	3 305	1 633
1609000	Powiat opolski	123 520	495 063 405,54	90,16	89,03	5 993	2 772
1610000	Powiat prudnicki	55 524	237 663 596,36	92,90	88,14	2 976	1 308
1611000	Powiat strzelecki	74 665	307 224 030,14	96,72	94,76	3 787	1 732
1661000	Powiat m.Opole	128 137	987 197 721,43	105,78	116,88	7 355	3 416
1801000	Powiat bieszczadzki	21 879	105 870 217,92	89,29	94,29	1 199	640
1802000	Powiat brzozowski	65 713	310 945 751,77	95,13	101,68	4 176	2 166
1803000	Powiat dębicki	135 471	606 606 168,88	92,75	98,69	8 497	4 123
1804000	Powiat jarosławski	120 714	539 481 963,13	92,33	95,76	7 014	3 400
1805000	Powiat jasielski	113 962	498 435 799,34	94,36	97,22	6 556	3 293
1806000	Powiat kolbuszowski	62 476	279 526 969,89	84,41	91,51	3 349	1 772
1807000	Powiat krośnieński	112 414	491 608 193,29	88,54	86,33	6 650	2 908
1808000	Powiat leżajski	69 562	312 185 447,88	90,16	90,77	3 970	1 939
1809000	Powiat lubaczowski	55 652	282 602 915,38	91,65	93,41	3 141	1 633
1810000	Powiat łańcucki	80 834	370 493 781,70	93,84	95,27	5 187	2 364
1811000	Powiat mielecki	136 673	645 081 583,17	93,66	101,27	8 294	4 211
1812000	Powiat niżański	66 843	291 330 579,47	86,19	89,17	3 416	1 791
1813000	Powiat przemyski	74 398	341 532 761,91	80,39	82,33	3 888	1 907
1814000	Powiat przeworski	78 458	383 822 352,95	93,99	94,96	4 868	2 315
1815000	Powiat ropczycko-sędziszow	74 374	372 297 098,69	93,74	94,85	4 829	2 325
1816000	Powiat rzeszowski	170 486	848 599 660,47	89,78	85,08	10 726	4 631
1817000	Powiat sanocki	94 758	421 982 060,63	89,47	91,21	5 334	2 576
1818000	Powiat stalowowolski	106 619	550 726 784,99	92,45	98,45	5 639	2 931
1819000	Powiat strzyżowski	61 558	282 736 934,49	94,23	93,33	3 574	1 847
1820000	Powiat tarnobrzeski	53 236	225 561 742,81	87,20	77,46	2 734	1 224
1821000	Powiat leski	26 562	132 508 088,68	88,91	91,56	1 428	662
1861000	Powiat m.Krosno	46 511	325 813 713,56	109,64	140,00	2 964	1 676
1862000	Powiat m.Przemyśl	61 251	401 722 533,77	105,54	139,70	3 708	2 270
1863000	Powiat m.Rzeszów	191 564	1 331 999 618,55	105,87	120,81	12 080	5 742
1864000	Powiat m.Tarnobrzeg	47 047	264 632 060,90	97,47	115,82	2 554	1 426

2001000	Powiat augustowski	58 367	266 358 440,93	90,69	99,30	3 210	1 726
2002000	Powiat białostocki	148 145	679 668 638,84	76,61	74,20	7 474	3 208
2003000	Powiat bielski	54 863	234 125 349,89	90,96	90,83	2 844	1 295
2004000	Powiat grajewski	47 518	229 750 240,80	93,03	92,07	2 766	1 370
2005000	Powiat hajnowski	43 143	181 118 472,11	90,92	96,82	1 899	1 011
2006000	Powiat kolneński	38 396	182 736 870,22	89,70	105,34	2 225	1 279
2007000	Powiat łomżyński	51 000	235 263 932,20	84,23	74,14	2 797	1 157
2008000	Powiat moniecki	40 712	186 036 809,74	86,01	95,20	1 986	1 106
2009000	Powiat sejneński	20 092	95 726 991,32	87,52	86,55	1 006	485
2010000	Powiat siemiatycki	44 689	203 581 296,87	92,09	88,07	2 175	1 050
2011000	Powiat sokólski	67 466	302 993 899,82	88,38	99,07	3 381	1 879
2012000	Powiat suwalski	35 792	178 737 019,35	82,72	84,05	1 962	993
2013000	Powiat wysokomazowiecki	57 248	269 211 943,33	91,33	88,16	3 323	1 432
2014000	Powiat zambrowski	43 816	189 583 813,59	93,63	92,96	2 628	1 184
2061000	Powiat m.Białystok	297 459	2 059 114 651,90	108,58	109,65	19 107	8 151
2062000	Powiat m.Łomża	63 000	386 730 250,15	101,89	117,94	3 803	1 964
2063000	Powiat m.Suwałki	69 827	451 802 429,35	101,39	110,39	4 673	2 231
2201000	Powiat bytowski	79 299	419 903 486,34	92,51	94,27	5 227	2 386
2202000	Powiat chojnicki	97 551	473 737 790,30	95,18	102,28	7 070	3 286
2203000	Powiat człuchowski	56 370	283 810 652,84	90,63	104,29	3 432	1 934
2204000	Powiat gdański	116 199	645 253 087,91	99,41	90,83	9 467	3 516
2205000	Powiat kartuski	136 619	776 011 550,10	96,96	101,70	11 849	5 502
2206000	Powiat kościerski	72 528	400 207 734,14	95,83	93,61	5 502	2 332
2207000	Powiat kwidzyński	83 291	383 770 883,97	89,96	95,29	5 540	2 681
2208000	Powiat lęborski	66 280	350 830 452,20	92,72	97,85	4 394	1 971
2209000	Powiat malborski	63 748	303 479 459,09	93,66	106,28	4 026	2 006
2210000	Powiat nowodworski	35 746	195 631 270,59	88,39	97,13	2 153	1 028
2211000	Powiat pucki	85 726	492 393 269,48	91,05	101,94	6 170	3 052
2212000	Powiat słupski	98 816	563 808 507,50	84,42	85,07	6 006	2 590
2213000	Powiat starogardzki	128 004	645 224 344,09	92,15	105,97	9 018	4 364
2214000	Powiat tczewski	115 876	526 389 531,07	94,02	99,56	8 013	3 500
2215000	Powiat wejherowski	215 908	1 136 302 800,05	97,68	108,82	16 979	7 794
2216000	Powiat sztumski	41 569	192 666 263,53	85,27	83,61	2 453	1 098
2261000	Powiat m.Gdańsk	466 631	3 276 985 537,89	98,92	108,97	27 363	12 216
2262000	Powiat m.Gdynia	246 309	1 605 977 090,39	94,50	110,19	13 266	6 756
2263000	Powiat m.Słupsk	91 007	552 234 995,13	100,48	118,92	5 158	2 639
2264000	Powiat m.Sopot	36 046	329 271 245,78	103,26	106,31	1 636	718

2401000	Powiat będziński	148 762	666 791 647,43	92,34	89,34	8 190	3 400
2402000	Powiat bielski	165 000	739 004 552,73	92,71	91,51	10 539	4 576
2403000	Powiat cieszyński	178 139	821 307 141,26	96,43	102,38	11 502	5 225
2404000	Powiat częstochowski	134 919	596 030 191,76	89,02	88,74	7 349	3 321
2405000	Powiat gliwicki	115 558	554 108 579,37	93,95	104,02	6 934	3 172
2406000	Powiat kłobucki	84 924	376 335 387,18	93,18	92,19	4 830	2 174
2407000	Powiat lubliniecki	76 593	349 783 085,14	97,35	92,69	4 672	2 030
2408000	Powiat mikołowski	98 373	503 524 745,72	95,74	97,58	6 563	2 790
2409000	Powiat myszkowski	71 119	321 552 139,77	95,50	97,65	3 994	1 853
2410000	Powiat pszczyński	111 202	530 400 947,14	97,87	105,32	7 950	3 463
2411000	Powiat raciborski	108 513	420 149 793,02	95,44	102,32	5 589	2 905
2412000	Powiat rybnicki	78 104	323 028 133,72	94,13	95,96	5 075	2 180
2413000	Powiat tarnogórski	139 833	635 177 084,79	95,21	92,76	8 116	3 423
2414000	Powiat bieruńsko-lędziński	59 760	297 198 722,21	94,79	94,01	3 926	1 642
2415000	Powiat wodzisławski	157 616	655 589 466,86	94,36	103,01	9 684	4 514
2416000	Powiat zawierciański	118 434	485 220 461,17	98,39	92,00	6 754	2 765
2417000	Powiat żywiecki	153 226	678 964 380,57	99,50	103,84	9 998	4 695
2461000	Powiat m.Bielsko-Biała	171 259	1 069 387 434,14	106,33	116,54	11 107	5 032
2462000	Powiat m.Bytom	166 795	891 584 914,58	92,08	98,94	8 608	4 070
2463000	Powiat m.Chorzów	108 434	663 721 620,70	96,76	118,32	6 455	3 221
2464000	Powiat m.Częstochowa	222 292	1 264 045 836,18	100,81	107,07	12 255	5 740
2465000	Powiat m.Dąbrowa Górnicza	120 259	787 780 599,65	98,51	102,47	6 911	2 913
2466000	Powiat m.Gliwice	179 806	1 212 988 376,76	96,98	114,69	10 019	4 854
2467000	Powiat m.Jastrzębie-Zdrój	89 128	452 405 275,01	95,54	94,70	5 268	2 318
2468000	Powiat m.Jaworzno	91 563	515 157 169,20	94,36	95,42	5 017	2 115
2469000	Powiat m.Katowice	294 510	1 958 524 153,88	103,97	100,71	15 705	6 470
2470000	Powiat m.Mysłowice	74 586	362 686 554,27	90,63	92,78	4 296	1 858
2471000	Powiat m.Piekary Śląskie	55 299	280 380 379,04	93,92	92,73	3 116	1 218
2472000	Powiat m.Ruda Śląska	138 000	770 390 352,25	92,61	94,33	8 032	3 439
2473000	Powiat m.Rybnik	138 696	930 844 849,80	95,33	108,60	8 580	4 019
2474000	Powiat m.Siemianowice Śląs	67 154	354 815 166,91	97,34	103,58	3 773	1 688
2475000	Powiat m.Sosnowiec	202 036	996 349 267,23	97,83	106,17	10 301	4 687
2476000	Powiat m.Świętochłowice	50 012	243 402 181,86	92,75	100,73	2 787	1 361
2477000	Powiat m.Tychy	127 831	825 555 390,66	96,46	105,81	7 682	3 401
2478000	Powiat m.Zabrze	173 374	871 596 094,18	95,56	106,18	9 195	4 440
2479000	Powiat m.Żory	62 456	334 748 367,64	95,19	103,04	4 198	1 914
2601000	Powiat buski	72 058	327 185 115,53	92,23	97,23	3 884	1 941

2602000	Powiat jędrzejowski	86 076	364 462 840,66	94,32	101,38	5 181	2 539
2603000	Powiat kazimierski	33 851	136 662 167,32	92,56	100,00	1 714	887
2604000	Powiat kielecki	210 694	1 012 633 247,70	90,38	93,51	12 820	5 955
2605000	Powiat konecki	80 648	327 860 145,05	92,62	101,55	4 266	2 220
2606000	Powiat opatowski	52 577	229 138 342,94	89,75	96,90	2 764	1 500
2607000	Powiat ostrowiecki	110 127	446 827 678,75	90,21	96,66	5 469	2 739
2608000	Powiat pińczowski	39 271	154 523 250,25	97,15	110,90	2 063	1 115
2609000	Powiat sandomierski	77 773	348 233 811,15	94,17	97,12	4 159	2 167
2610000	Powiat skarżyski	74 817	280 113 106,06	90,36	95,80	3 533	1 723
2611000	Powiat starachowicki	90 377	389 622 080,92	92,66	101,01	4 895	2 428
2612000	Powiat staszowski	72 167	336 240 238,65	94,33	95,22	4 229	1 927
2613000	Powiat włoszczowski	45 336	195 951 064,22	94,72	96,25	2 622	1 305
2661000	Powiat m.Kielce	195 774	1 275 112 674,57	101,69	120,95	11 023	5 484
2801000	Powiat bartoszycki	57 916	269 506 200,53	88,83	100,55	3 291	1 658
2802000	Powiat braniewski	41 391	183 490 118,30	87,22	95,34	2 175	1 071
2803000	Powiat działdowski	65 442	290 823 267,86	93,58	97,29	4 296	1 977
2804000	Powiat elbląski	57 549	268 287 814,54	78,63	92,60	3 079	1 755
2805000	Powiat ełcki	91 359	430 420 218,31	88,38	94,90	5 618	2 697
2806000	Powiat giżycki	56 754	275 338 213,22	93,71	102,10	3 433	1 646
2807000	Powiat iławski	93 020	449 139 566,35	94,14	98,38	6 313	2 944
2808000	Powiat kętrzyński	62 924	282 232 874,50	89,73	95,61	3 507	1 727
2809000	Powiat lidzbarski	41 512	206 919 468,11	88,60	93,83	2 374	1 062
2810000	Powiat mrągowski	50 080	233 153 718,96	90,39	95,07	2 924	1 442
2811000	Powiat nidzicki	33 068	156 017 584,22	90,24	98,79	1 944	1 041
2812000	Powiat nowomiejski	43 997	231 006 096,78	94,89	100,20	3 138	1 419
2813000	Powiat olecki	34 433	169 704 832,92	91,44	96,91	2 159	1 069
2814000	Powiat olsztyński	126 018	616 688 610,07	80,28	74,80	7 051	2 904
2815000	Powiat ostródzki	104 780	503 715 725,34	91,10	95,61	6 556	3 048
2816000	Powiat piski	56 570	258 056 168,37	86,92	94,92	3 253	1 689
2817000	Powiat szczycieński	69 885	318 093 508,63	91,20	93,32	4 367	2 026
2818000	Powiat gołdapski	26 908	138 255 664,03	88,78	88,30	1 697	757
2819000	Powiat węgorzewski	22 873	94 685 432,50	88,46	89,05	1 234	557
2861000	Powiat m.Elbląg	120 142	636 960 971,43	94,15	102,48	6 766	3 273
2862000	Powiat m.Olsztyn	172 362	1 200 980 666,45	106,46	128,99	11 063	5 540
3001000	Powiat chodzieski	47 229	228 248 783,87	98,59	109,24	3 244	1 641
3002000	Powiat czarnkowsko-trzcian	87 420	397 169 094,41	95,51	101,75	5 967	2 724
3003000	Powiat gnieźnieński	145 317	672 282 764,63	96,29	100,29	10 030	4 388

3004000	Powiat gostyński	76 011	334 389 809,45	99,20	99,48	5 185	2 242
3005000	Powiat grodziski	51 972	241 743 555,10	94,99	94,40	3 710	1 638
3006000	Powiat jarociński	71 686	312 853 315,43	98,55	107,96	4 893	2 289
3007000	Powiat kaliski	83 030	382 398 234,38	88,27	92,69	4 850	2 398
3008000	Powiat kępiński	56 486	288 032 379,17	98,50	96,02	3 831	1 687
3009000	Powiat kolski	87 216	394 634 121,93	93,73	103,33	5 372	2 596
3010000	Powiat koniński	129 966	615 922 990,92	89,52	88,14	8 008	3 703
3011000	Powiat kościański	79 230	378 066 016,87	98,45	103,96	5 280	2 269
3012000	Powiat krotoszyński	77 488	327 685 398,02	97,41	104,86	5 242	2 454
3013000	Powiat leszczyński	56 485	272 824 116,82	87,36	89,53	3 855	1 714
3014000	Powiat międzychodzki	36 982	186 884 767,50	96,04	94,30	2 472	1 078
3015000	Powiat nowotomyski	75 363	354 997 287,98	98,73	100,55	5 525	2 338
3016000	Powiat obornicki	59 858	270 657 904,22	95,53	101,10	4 312	1 941
3017000	Powiat ostrowski	161 519	702 829 542,89	96,58	97,85	10 191	4 552
3018000	Powiat ostrzeszowski	55 403	250 112 511,91	99,08	97,74	3 861	1 608
3019000	Powiat pilski	136 621	628 828 859,00	96,10	98,99	8 827	4 151
3020000	Powiat pleszewski	63 147	288 382 067,07	92,64	102,14	4 022	2 037
3021000	Powiat poznański	390 308	2 067 915 371,69	91,01	90,05	29 410	11 655
3022000	Powiat rawicki	60 405	271 099 969,97	96,23	104,54	4 279	2 040
3023000	Powiat słupecki	59 371	264 775 869,19	95,80	95,97	3 808	1 670
3024000	Powiat szamotulski	91 185	420 666 445,26	96,67	115,15	6 409	3 293
3025000	Powiat średzki	58 456	272 110 954,38	98,47	102,39	4 092	1 723
3026000	Powiat śremski	61 325	267 465 695,43	99,64	98,08	4 370	1 830
3027000	Powiat turecki	84 101	404 416 498,18	96,34	98,47	5 521	2 534
3028000	Powiat wągrowiecki	70 274	327 111 332,80	95,96	107,73	5 017	2 422
3029000	Powiat wolsztyński	57 326	253 396 262,97	99,34	109,41	4 134	2 057
3030000	Powiat wrzesiński	77 667	351 272 567,77	98,10	96,37	5 508	2 167
3031000	Powiat złotowski	69 606	306 023 180,07	92,02	94,31	4 462	2 106
3061000	Powiat m.Kalisz	100 975	611 344 009,75	104,42	109,69	6 281	2 925
3062000	Powiat m.Konin	74 151	513 228 887,02	111,30	127,57	4 547	2 273
3063000	Powiat m.Leszno	63 952	384 014 865,99	110,23	124,63	4 503	2 306
3064000	Powiat m.Poznań	536 438	3 698 759 702,12	108,74	138,41	33 004	16 898
3201000	Powiat białogardzki	47 846	229 453 671,33	91,76	92,77	2 902	1 321
3202000	Powiat choszczeński	48 602	221 045 451,76	88,66	86,45	2 796	1 275
3203000	Powiat drawski	57 397	281 506 157,99	89,42	102,76	3 327	1 671
3204000	Powiat goleniowski	82 540	378 671 936,39	93,04	95,99	5 216	2 456
3205000	Powiat gryficki	60 617	322 493 962,48	89,54	99,83	3 577	1 782

3206000	Powiat gryfiński	82 530	377 204 687,75	89,98	91,61	4 874	2 197
3207000	Powiat kamieński	47 227	262 576 687,92	89,46	82,15	2 490	1 074
3208000	Powiat kołobrzeski	79 547	457 653 286,12	94,75	101,06	4 488	2 152
3209000	Powiat koszaliński	66 355	355 191 971,00	76,05	81,43	3 339	1 576
3210000	Powiat myśliborski	66 264	316 976 607,92	91,53	99,53	3 959	2 005
3211000	Powiat policki	79 476	417 171 483,24	78,70	60,32	4 812	1 568
3212000	Powiat pyrzycki	39 639	168 414 204,16	91,05	100,29	2 372	1 127
3213000	Powiat sławieński	56 409	325 959 289,08	87,97	91,84	3 231	1 519
3214000	Powiat stargardzki	120 112	561 618 541,48	92,06	101,13	7 133	3 361
3215000	Powiat szczecinecki	77 965	352 007 634,17	89,06	95,46	4 368	2 145
3216000	Powiat świdwiński	47 242	222 146 926,29	88,37	91,55	2 619	1 296
3217000	Powiat wałecki	53 462	242 537 388,99	89,71	100,72	3 052	1 680
3218000	Powiat łobeski	37 104	174 804 582,96	89,82	88,65	2 178	957
3261000	Powiat m.Koszalin	107 321	611 075 958,77	107,92	121,84	6 625	3 140
3262000	Powiat m.Szczecin	402 465	2 288 818 260,90	100,85	110,01	22 684	10 749
3263000	Powiat m.Świnoujście	40 910	375 707 857,62	97,11	107,84	2 152	998

Code	Powiat	Employment (of the population aged 15-64) in:				Working age population (production population)	Uneployment	Feminization ratio	Women
		agriculture	industry	services	financial sector				
		in person	in person	in person	in person	in persons	in persons	females per 100 males	in persons
0201000	Powiat bolesławiecki	4 377	10 497	4 720	414	55 216	1 283	105,95	46 402
0202000	Powiat dzierżoniowski	2 360	8 336	2 629	480	60 894	1 754	109,78	53 082
0203000	Powiat głogowski	2 054	7 144	4 617	655	53 452	2 602	105,38	45 943
0204000	Powiat górowski	3 673	1 093	652	87	21 605	1 671	101,80	17 748
0205000	Powiat jaworski	2 889	3 163	1 162	276	31 002	1 956	104,26	25 799
0206000	Powiat jeleniogórski	1 414	4 460	2 470	164	39 282	1 734	106,87	32 988
0207000	Powiat kamiennogórski	1 789	3 669	1 041	271	26 650	825	104,82	22 324
0208000	Powiat kłodzki	5 339	7 569	5 116	772	95 946	5 717	107,07	82 387
0209000	Powiat legnicki	3 391	5 240	2 019	171	34 180	1 921	103,83	28 183
0210000	Powiat lubański	2 706	3 447	1 826	269	33 469	1 234	105,29	28 054
0211000	Powiat lubiński	2 584	9 701	6 312	567	63 004	1 765	106,94	54 909
0212000	Powiat lwówecki	2 820	2 180	1 085	183	28 450	1 645	105,19	23 612
0213000	Powiat milicki	2 301	2 757	1 064	136	22 171	894	102,19	18 747
0214000	Powiat oleśnicki	5 550	10 817	3 956	443	65 414	1 999	104,82	54 790
0215000	Powiat oławski	2 642	17 306	3 060	419	46 388	2 030	105,29	39 285
0216000	Powiat polkowicki	2 691	21 692	3 709	492	39 404	1 689	102,26	31 843
0217000	Powiat strzeliński	3 194	2 786	1 233	190	26 948	1 538	101,90	22 092
0218000	Powiat średzki	2 859	7 742	2 136	99	35 851	1 171	102,21	27 431
0219000	Powiat świdnicki	4 572	19 771	5 748	623	95 522	3 627	107,19	81 543
0220000	Powiat trzebnicki	5 294	6 404	3 490	234	52 304	1 734	103,34	43 140
0221000	Powiat wałbrzyski	1 277	1 722	1 319	205	34 260	1 717	107,27	28 984
0222000	Powiat wołowski	2 710	4 282	1 306	127	28 955	1 902	104,37	24 031
0223000	Powiat wrocławski	6 196	18 379	20 415	440	90 740	1 334	104,27	74 558
0224000	Powiat ząbkowicki	4 450	4 243	2 277	298	40 036	2 416	105,50	33 589
0225000	Powiat zgorzelecki	1 917	7 240	4 451	535	55 336	1 437	105,60	46 227
0226000	Powiat złotoryjski	2 785	2 439	831	141	27 337	2 023	103,95	22 359
0261000	Powiat m.Jelenia Góra	374	8 049	4 867	616	45 726	1 209	115,38	42 577
0262000	Powiat m.Legnica	329	12 913	7 512	1 274	59 487	2 279	111,26	52 534
0264000	Powiat m.Wrocław	1 967	51 581	85 806	20 504	379 390	7 247	114,13	341 458
0265000	Powiat m.Wałbrzych od 201	308	12 787	5 804	815	66 495	2 489	112,58	59 628
0401000	Powiat aleksandrowski	4 506	2 522	1 482	298	33 705	2 564	105,77	28 412

0402000	Powiat brodnicki	6 185	9 989	3 290	397	48 738	2 262	102,01	39 841
0403000	Powiat bydgoski	4 791	11 523	6 481	528	73 917	2 370	101,98	59 237
0404000	Powiat chełmiński	3 682	4 319	1 278	187	32 349	2 193	104,11	26 622
0405000	Powiat golubsko-dobrzyński	4 925	4 102	2 110	195	28 069	1 892	101,54	22 743
0406000	Powiat grudziądzki	4 728	2 735	912	109	25 204	1 909	99,77	20 112
0407000	Powiat inowrocławski	7 677	14 856	6 964	1 346	99 493	7 581	106,41	82 819
0408000	Powiat lipnowski	7 070	2 122	1 970	314	41 327	3 923	101,09	33 210
0409000	Powiat mogileński	3 876	3 228	2 180	367	28 420	1 840	104,28	23 390
0410000	Powiat nakielski	5 143	4 638	3 645	604	53 629	3 290	101,71	43 661
0411000	Powiat radziejowski	5 628	1 191	1 352	154	25 325	2 624	102,15	20 574
0412000	Powiat rypiński	4 750	3 478	1 184	178	26 724	1 896	102,36	22 169
0413000	Powiat sępoleński	3 133	3 329	1 189	389	25 220	1 863	100,27	20 601
0414000	Powiat świecki	5 261	10 219	4 154	669	61 199	2 592	102,87	50 342
0415000	Powiat toruński	7 010	7 343	3 672	342	67 201	3 845	102,95	54 244
0416000	Powiat tucholski	4 619	3 940	1 614	221	29 530	2 003	100,10	24 199
0417000	Powiat wąbrzeski	3 786	2 711	1 014	137	21 164	1 575	103,19	17 477
0418000	Powiat włocławski	11 817	3 864	2 375	166	54 183	5 179	101,87	43 576
0419000	Powiat żniński	5 693	3 859	2 765	750	43 023	2 628	102,29	35 605
0461000	Powiat m.Bydgoszcz	786	37 154	32 508	7 504	205 548	5 982	112,55	185 424
0462000	Powiat m.Grudziądz	522	9 689	5 312	758	56 123	3 233	110,01	49 788
0463000	Powiat m.Toruń	482	16 417	21 162	3 565	120 698	4 463	115,05	108 110
0464000	Powiat m.Włocławek	617	14 736	6 000	1 392	65 467	4 948	112,52	58 664
0601000	Powiat bialski	19 072	3 544	5 320	397	68 230	4 442	101,23	56 037
0602000	Powiat biłgorajski	24 488	6 021	2 138	343	62 269	2 380	102,85	51 430
0603000	Powiat chełmski	16 735	1 588	1 264	143	48 756	4 071	102,62	39 620
0604000	Powiat hrubieszowski	13 651	1 174	1 430	221	38 702	3 564	103,84	32 449
0605000	Powiat janowski	13 511	1 889	832	127	28 235	2 019	101,91	23 246
0606000	Powiat krasnostawski	13 729	3 155	1 270	289	38 005	3 299	105,43	32 808
0607000	Powiat kraśnicki	19 675	5 662	2 464	335	58 112	4 412	105,77	49 368
0608000	Powiat lubartowski	15 205	5 001	2 570	233	53 915	3 976	104,77	45 429
0609000	Powiat lubelski	26 452	5 288	4 419	324	95 106	3 603	104,19	78 777
0610000	Powiat łęczyński	7 810	9 519	1 685	221	36 268	1 446	103,84	29 270
0611000	Powiat łukowski	17 768	5 998	4 895	366	64 696	2 037	101,04	54 002
0612000	Powiat opolski	14 218	2 198	1 377	194	78 807	2 468	104,81	30 564
0613000	Powiat parczewski	6 811	1 569	775	111	20 992	1 032	101,58	17 614
0614000	Powiat puławski	13 934	9 860	4 066	722	66 938	3 071	108,71	59 255
0615000	Powiat radzyński	11 377	3 042	1 615	179	36 071	1 771	100,36	29 692

0616000	Powiat rycki	8 154	3 432	2 034	152	33 770	1 787	100,77	28 191
0617000	Powiat świdnicki	6 733	5 681	2 562	301	42 746	2 414	107,90	37 389
0618000	Powiat tomaszowski	18 192	3 737	1 984	278	51 353	2 457	102,92	42 460
0619000	Powiat włodawski	5 247	1 341	596	118	23 464	2 229	102,80	19 601
0620000	Powiat zamojski	24 701	4 242	1 728	111	65 840	4 028	102,94	54 242
0661000	Powiat m.Biała Podlaska	1 362	3 406	4 370	549	35 245	2 475	108,89	29 897
0662000	Powiat m.Chełm	1 623	3 020	3 897	595	38 010	2 613	112,98	33 245
0663000	Powiat m.Lublin	4 940	24 237	34 318	7 934	201 034	10 050	116,87	183 051
0664000	Powiat m.Zamość	1 750	3 552	4 677	739	38 832	2 805	112,31	33 757
0801000	Powiat gorzowski	4 662	8 363	2 793	241	45 148	1 118	101,50	36 041
0802000	Powiat krośnieński	3 074	3 326	2 011	196	34 304	1 619	103,42	28 087
0803000	Powiat międzyrzecki	2 516	4 645	2 212	276	35 636	2 462	102,68	29 396
0804000	Powiat nowosolski	2 633	8 067	2 499	330	51 929	2 334	105,25	44 424
0805000	Powiat słubicki	1 463	3 105	4 318	290	29 647	413	104,25	24 040
0806000	Powiat strzelecko-drezdenec	3 472	3 980	1 581	253	30 083	1 902	102,45	24 982
0807000	Powiat sulęciński	1 958	2 386	1 544	141	21 899	740	101,24	17 757
0808000	Powiat świebodziński	1 894	9 766	3 467	234	34 349	1 109	104,19	28 493
0809000	Powiat zielonogórski	4 035	6 258	3 284	135	47 124	1 722	102,40	38 325
0810000	Powiat żagański	2 730	4 147	2 623	374	49 005	1 957	105,11	40 783
0811000	Powiat żarski	3 386	9 719	4 062	310	59 728	1 943	106,05	49 860
0812000	Powiat wschowski	2 670	2 753	1 402	188	23 905	1 119	102,60	19 783
0861000	Powiat m.Gorzów Wielkopo	1 052	14 196	9 259	1 609	72 908	1 482	110,34	65 006
0862000	Powiat m.Zielona Góra	397	12 145	13 829	1 827	82 939	2 281	110,74	73 723
1001000	Powiat bełchatowski	7 007	20 015	5 291	1 044	70 696	3 047	104,21	57 663
1002000	Powiat kutnowski	8 663	10 444	4 854	466	58 378	3 671	107,98	50 514
1003000	Powiat łaski	5 685	3 119	1 694	187	30 542	1 453	105,45	25 716
1004000	Powiat łęczycki	9 088	2 692	1 578	157	30 201	1 532	105,71	25 768
1005000	Powiat łowicki	12 959	5 164	3 009	355	47 174	1 818	106,56	40 557
1006000	Powiat łódzki wschodni	3 838	5 831	4 341	470	43 475	1 984	108,32	37 285
1007000	Powiat opoczyński	10 257	5 533	3 059	202	46 393	1 761	102,06	38 703
1008000	Powiat pabianicki	4 052	12 110	5 475	967	70 580	3 174	112,87	63 250
1009000	Powiat pajęczański	7 728	3 299	1 444	118	31 643	1 530	102,21	26 081
1010000	Powiat piotrkowski	13 959	3 230	5 317	109	55 229	1 851	102,97	46 325
1011000	Powiat poddębicki	7 559	2 990	948	175	24 944	1 142	102,07	20 814
1012000	Powiat radomszczański	9 426	9 616	5 247	488	68 546	2 702	104,11	57 799
1013000	Powiat rawski	8 305	2 960	2 434	234	29 366	823	102,80	24 741
1014000	Powiat sieradzki	18 794	6 889	5 406	509	72 127	3 201	104,97	60 553

1015000	Powiat skierniewicki	8 821	2 175	1 183	46	22 955	610	101,20	19 211
1016000	Powiat tomaszowski	9 297	10 620	6 494	628	69 238	3 624	108,58	61 040
1017000	Powiat wieluński	10 602	6 881	2 779	347	46 568	2 183	103,89	39 081
1018000	Powiat wieruszowski	4 997	5 503	1 311	164	25 964	853	101,81	21 296
1019000	Powiat zduńskowolski	3 080	8 527	2 773	506	40 380	1 847	106,77	34 476
1020000	Powiat zgierski	6 935	15 007	9 545	752	98 552	4 292	110,67	87 158
1021000	Powiat brzeziński	3 588	1 349	1 082	109	18 677	800	105,76	15 877
1061000	Powiat m.Łódź	2 011	66 039	66 836	17 076	391 389	19 251	119,41	372 957
1062000	Powiat m.Piotrków Trybuna	851	8 375	4 996	733	42 952	1 905	113,59	39 178
1063000	Powiat m.Skierniewice	728	4 318	1 879	453	28 293	982	108,94	25 120
1201000	Powiat bocheński	11 983	9 318	3 290	217	65 195	1 863	102,53	53 922
1202000	Powiat brzeski	13 298	5 584	2 715	270	58 019	1 912	101,68	46 991
1203000	Powiat chrzanowski	2 409	12 009	5 297	659	75 976	3 303	106,59	64 691
1204000	Powiat dąbrowski	11 493	2 044	1 330	125	37 850	2 550	100,51	29 751
1205000	Powiat gorlicki	18 788	6 062	4 779	286	66 899	2 478	101,83	55 047
1206000	Powiat krakowski	19 838	21 427	18 545	744	170 177	4 519	104,30	141 491
1207000	Powiat limanowski	23 073	7 135	3 927	406	80 717	4 198	99,77	65 686
1208000	Powiat miechowski	10 570	1 004	2 069	105	29 138	945	103,08	24 941
1209000	Powiat myślenicki	13 909	9 417	4 671	302	78 907	1 312	101,63	64 028
1210000	Powiat nowosądecki	27 274	9 912	6 600	453	133 974	6 154	100,70	108 467
1211000	Powiat nowotarski	21 492	8 404	6 851	552	119 260	4 036	104,39	97 811
1212000	Powiat olkuski	8 293	12 998	5 322	1 332	67 395	3 469	105,83	57 603
1213000	Powiat oświęcimski	6 202	14 119	6 078	570	93 035	2 900	105,65	78 981
1214000	Powiat proszowicki	9 935	824	1 609	122	26 708	1 139	102,94	22 077
1215000	Powiat suski	10 215	6 624	2 711	179	51 918	1 560	101,60	42 461
1216000	Powiat tarnowski	31 681	7 754	6 602	204	127 040	5 088	101,59	101 581
1217000	Powiat tatrzański	6 655	1 127	4 019	381	41 360	2 780	107,31	35 274
1218000	Powiat wadowicki	11 458	14 129	6 384	530	98 521	3 160	103,97	81 624
1219000	Powiat wielicki	6 246	12 817	6 316	416	78 389	2 221	104,84	65 040
1261000	Powiat m.Kraków	4 471	67 296	109 949	21 637	459 543	11 467	114,27	411 204
1262000	Powiat m.Nowy Sącz	1 077	12 015	10 532	1 133	50 467	1 502	108,90	43 736
1263000	Powiat m.Tarnów	1 382	14 404	9 261	1 064	64 708	2 933	111,68	57 540
1401000	Powiat białobrzeski	6 564	1 063	1 073	110	20 443	1 079	97,96	16 603
1402000	Powiat ciechanowski	5 613	7 032	4 039	563	54 807	3 378	103,43	45 590
1403000	Powiat garwoliński	12 565	8 197	4 105	208	65 782	3 902	101,22	54 827
1404000	Powiat gostyniński	4 764	1 985	1 414	179	27 583	2 524	105,24	23 179
1405000	Powiat grodziski	2 109	6 807	7 007	521	55 693	1 046	108,61	48 987

1406000	Powiat grójecki	17 695	8 258	4 115	546	58 977	953	103,45	50 060
1407000	Powiat kozienicki	7 807	5 206	1 149	155	36 501	2 634	101,49	30 459
1408000	Powiat legionowski	1 552	3 977	4 540	854	69 263	2 331	107,24	60 428
1409000	Powiat lipski	8 541	1 431	626	92	20 563	1 597	101,48	17 233
1410000	Powiat łosicki	8 525	1 198	1 296	119	18 465	879	100,90	15 581
1411000	Powiat makowski	6 731	1 499	1 273	117	27 302	3 019	100,09	22 634
1412000	Powiat miński	8 122	9 020	6 554	554	92 461	2 554	105,09	78 821
1413000	Powiat mławski	7 911	7 022	2 961	347	45 229	1 727	103,95	37 259
1414000	Powiat nowodworski	3 459	8 074	5 064	507	22 357	1 901	101,36	40 575
1415000	Powiat ostrołęcki	15 971	3 034	2 174	124	56 163	3 322	97,47	43 800
1416000	Powiat ostrowski	9 642	5 046	2 798	340	44 341	2 478	101,93	36 739
1417000	Powiat otwocki	3 772	11 484	6 729	853	73 171	2 008	108,71	64 605
1418000	Powiat piaseczyński	5 262	12 853	18 087	1 784	110 151	3 487	108,67	96 318
1419000	Powiat płocki	14 132	3 959	3 161	223	69 479	4 338	101,66	56 001
1420000	Powiat płoński	12 117	4 182	3 180	396	53 356	3 622	103,23	44 399
1421000	Powiat pruszkowski	1 631	13 373	18 539	1 460	96 393	2 541	110,28	86 134
1422000	Powiat przasnyski	8 248	2 429	1 893	177	32 051	2 027	99,81	26 387
1423000	Powiat przysuski	8 609	1 250	684	114	25 599	3 515	101,24	21 090
1424000	Powiat pułtuski	6 569	2 744	1 345	230	31 754	2 555	102,28	26 197
1425000	Powiat radomski	17 757	7 050	3 277	253	94 552	9 489	100,72	76 333
1426000	Powiat siedlecki	14 129	4 989	2 379	119	49 800	1 729	98,65	40 399
1427000	Powiat sierpecki	5 201	2 653	1 503	256	32 348	3 106	103,07	26 492
1428000	Powiat sochaczewski	7 171	5 939	6 652	407	51 521	2 084	105,17	43 624
1429000	Powiat sokołowski	8 354	4 248	2 513	257	32 329	1 326	101,98	27 374
1430000	Powiat szydłowiecki	3 419	1 449	797	71	24 679	3 541	100,44	19 976
1432000	Powiat warszawski zachodn	3 204	11 910	18 851	813	69 734	1 240	107,90	60 727
1433000	Powiat węgrowski	10 323	3 710	1 998	210	39 795	1 624	100,19	33 118
1434000	Powiat wołomiński	6 392	13 127	12 416	1 249	149 095	5 951	107,45	127 084
1435000	Powiat wyszkowski	8 530	5 284	4 677	258	45 474	1 327	101,99	37 397
1436000	Powiat zwoleński	6 975	1 210	950	83	22 236	1 588	100,72	18 229
1437000	Powiat żuromiński	7 549	1 067	817	129	23 879	2 480	102,65	19 727
1438000	Powiat żyrardowski	3 683	3 681	4 843	538	44 913	2 765	109,17	39 587
1461000	Powiat m.Ostrołęka	602	6 003	3 586	652	31 341	2 302	109,49	27 315
1462000	Powiat m.Płock	888	17 392	10 289	1 425	70 626	4 269	112,11	63 425
1463000	Powiat m.Radom	2 262	18 616	13 857	1 485	126 834	11 123	110,69	111 918
1464000	Powiat m.Siedlce	1 127	7 708	6 489	700	45 384	1 802	111,38	41 033
1465000	Powiat m. st. Warszawa	5 369	129 285	306 758	125 297	1 027 633	19 382	117,45	960 312

1601000	Powiat brzeski	3 384	5 863	3 402	421	55 353	2 479	105,26	46 295
1602000	Powiat głubczycki	3 834	2 796	948	193	28 204	1 472	104,78	23 477
1603000	Powiat kędzierzyńsko-kozie	3 665	9 363	4 794	416	58 399	2 705	107,24	48 893
1604000	Powiat kluczborski	3 617	4 441	2 334	299	41 623	1 561	105,02	33 725
1605000	Powiat krapkowicki	3 636	12 010	2 224	259	40 519	1 435	106,86	33 031
1606000	Powiat namysłowski	2 368	3 189	965	203	26 314	1 026	104,89	21 853
1607000	Powiat nyski	6 704	7 746	4 513	583	84 489	3 250	105,77	70 393
1608000	Powiat oleski	7 294	5 914	2 070	168	40 411	1 027	104,91	33 075
1609000	Powiat opolski	5 718	8 905	3 574	199	36 179	2 226	106,75	63 777
1610000	Powiat prudnicki	4 092	2 291	1 819	260	34 260	1 655	107,93	28 821
1611000	Powiat strzelecki	4 332	7 015	2 954	432	47 355	1 273	105,45	38 323
1661000	Powiat m.Opole	544	17 188	14 491	2 097	76 067	2 554	112,10	67 724
1801000	Powiat bieszczadzki	2 790	552	719	103	13 917	1 175	103,24	11 114
1802000	Powiat brzozowski	12 382	2 439	1 589	116	40 573	4 415	102,12	33 201
1803000	Powiat dębicki	17 020	15 226	6 730	390	84 371	3 452	102,44	68 551
1804000	Powiat jarosławski	16 300	9 866	4 370	756	75 146	6 551	104,79	61 769
1805000	Powiat jasielski	19 101	10 003	3 370	360	71 611	5 179	103,36	57 923
1806000	Powiat kolbuszowski	9 364	2 975	1 951	122	39 989	1 972	100,51	31 317
1807000	Powiat krośnieński	14 645	5 087	2 788	135	69 095	2 187	103,27	57 111
1808000	Powiat leżajski	10 128	3 373	1 910	184	43 479	3 686	102,39	35 192
1809000	Powiat lubaczowski	11 804	1 520	1 115	210	35 030	2 125	101,43	28 023
1810000	Powiat łańcucki	9 950	4 761	2 834	200	49 110	3 482	103,76	41 163
1811000	Powiat mielecki	14 626	23 276	5 959	549	84 342	2 895	102,91	69 316
1812000	Powiat niżański	8 238	2 981	1 374	104	43 071	3 868	102,19	33 784
1813000	Powiat przemyski	14 381	1 217	2 024	95	47 500	3 691	99,40	37 087
1814000	Powiat przeworski	13 799	4 876	1 855	266	48 742	4 085	102,98	39 804
1815000	Powiat ropczycko-sędziszow	8 216	5 961	2 305	199	45 964	3 426	102,47	37 640
1816000	Powiat rzeszowski	26 041	13 289	8 149	297	106 004	6 405	102,58	86 330
1817000	Powiat sanocki	10 784	11 892	3 362	567	58 369	2 822	104,12	48 336
1818000	Powiat stalowowolski	7 176	14 637	4 627	709	65 405	2 417	106,05	54 875
1819000	Powiat strzyżowski	11 954	2 584	1 507	120	38 565	3 730	101,72	31 041
1820000	Powiat tarnobrzeski	8 104	6 827	1 478	96	33 501	1 526	103,38	27 061
1821000	Powiat leski	4 157	532	763	77	16 599	1 923	102,47	13 443
1861000	Powiat m.Krosno	459	10 528	4 593	472	27 411	789	111,56	24 526
1862000	Powiat m.Przemyśl	1 392	3 640	4 572	647	36 519	3 088	113,12	32 511
1863000	Powiat m.Rzeszów	3 378	24 122	23 698	4 407	116 565	6 571	110,87	100 719
1864000	Powiat m.Tarnobrzeg	1 797	2 551	2 104	416	28 453	1 473	108,99	24 535

2001000	Powiat augustowski	6 496	4 250	1 954	214	36 229	2 157	104,16	29 778
2002000	Powiat białostocki	14 536	10 166	4 803	319	92 276	4 419	102,74	75 072
2003000	Powiat bielski	8 804	5 762	1 766	278	32 388	1 310	101,95	27 696
2004000	Powiat grajewski	5 731	2 852	1 370	149	29 914	2 389	101,48	23 934
2005000	Powiat hajnowski	4 547	3 849	1 652	145	24 900	1 297	106,22	22 222
2006000	Powiat kolneński	8 457	1 087	722	105	24 459	2 289	99,65	19 164
2007000	Powiat łomżyński	11 123	2 322	1 033	140	32 196	1 361	97,67	25 200
2008000	Powiat moniecki	8 663	966	511	123	25 616	1 137	101,39	20 496
2009000	Powiat sejneński	4 528	282	408	42	12 400	1 090	100,22	10 057
2010000	Powiat siemiatycki	7 520	1 900	1 282	125	27 370	1 189	103,46	22 725
2011000	Powiat sokólski	12 651	2 834	1 852	208	41 692	3 045	102,13	34 088
2012000	Powiat suwalski	8 034	1 572	761	54	22 241	574	95,79	17 511
2013000	Powiat wysokomazowiecki	13 870	3 851	1 655	254	34 753	1 567	98,12	28 352
2014000	Powiat zambrowski	5 753	3 334	2 028	197	27 223	1 357	101,70	22 093
2061000	Powiat m.Białystok	2 958	18 926	25 093	4 140	181 826	8 001	113,05	157 838
2062000	Powiat m.Łomża	1 175	3 689	3 817	490	39 154	2 062	109,76	32 966
2063000	Powiat m.Suwałki	1 081	7 507	4 569	773	43 796	1 542	109,34	36 471
2201000	Powiat bytowski	5 374	7 024	2 327	405	49 046	2 907	100,75	39 798
2202000	Powiat chojnicki	5 675	9 262	4 988	482	59 205	3 213	102,45	49 365
2203000	Powiat człuchowski	3 364	4 014	1 797	394	34 771	1 883	102,40	28 519
2204000	Powiat gdański	3 037	8 731	8 764	506	72 497	1 718	103,73	59 164
2205000	Powiat kartuski	9 613	11 761	6 024	260	82 215	1 490	100,29	68 410
2206000	Powiat kościerski	5 621	5 509	2 965	216	43 962	1 829	99,99	36 263
2207000	Powiat kwidzyński	3 942	11 324	3 036	406	51 718	1 720	101,68	41 993
2208000	Powiat lęborski	1 405	4 445	2 381	900	40 553	1 899	103,26	33 671
2209000	Powiat malborski	1 409	2 656	2 167	433	39 035	2 203	105,00	32 652
2210000	Powiat nowodworski	1 813	1 548	1 055	122	48 508	1 500	104,80	17 994
2211000	Powiat pucki	2 107	5 554	3 934	288	52 907	1 950	101,83	43 251
2212000	Powiat słupski	4 796	10 907	2 938	233	61 745	2 289	100,06	49 424
2213000	Powiat starogardzki	5 812	12 242	5 128	703	78 954	2 508	102,85	64 902
2214000	Powiat tczewski	2 650	16 520	4 674	418	70 793	2 815	103,49	58 932
2215000	Powiat wejherowski	4 605	11 781	8 067	845	131 819	3 999	102,13	109 093
2216000	Powiat sztumski	2 013	2 548	718	210	26 109	1 113	101,17	20 905
2261000	Powiat m.Gdańsk	1 255	35 317	55 858	12 233	272 935	6 552	110,97	245 444
2262000	Powiat m.Gdynia	693	16 443	26 746	5 008	143 177	2 779	111,73	129 979
2263000	Powiat m.Słupsk	261	9 449	6 067	1 564	53 536	1 369	112,40	48 160
2264000	Powiat m.Sopot	85	1 777	3 739	1 742	20 114	346	115,35	19 308

2401000	Powiat będziński	3 558	9 621	7 648	863	87 908	3 713	110,07	77 946
2402000	Powiat bielski	7 718	19 552	7 457	659	100 490	1 942	105,00	84 513
2403000	Powiat cieszyński	9 951	16 696	8 669	692	106 945	3 330	105,91	91 628
2404000	Powiat częstochowski	11 658	8 572	4 067	171	83 442	3 123	104,10	68 815
2405000	Powiat gliwicki	4 811	10 954	4 900	465	71 212	1 818	106,17	59 507
2406000	Powiat kłobucki	9 093	7 582	2 581	132	52 725	2 448	102,73	43 034
2407000	Powiat lubliniecki	3 890	6 953	2 865	205	47 652	1 686	103,47	38 950
2408000	Powiat mikołowski	1 378	15 812	5 348	580	59 867	1 290	105,67	50 542
2409000	Powiat myszkowski	3 990	7 066	2 429	161	42 794	1 589	107,12	36 782
2410000	Powiat pszczyński	5 080	14 474	4 997	557	69 717	1 400	104,56	56 840
2411000	Powiat raciborski	4 431	13 127	5 010	618	68 429	1 657	108,19	56 390
2412000	Powiat rybnicki	2 627	4 550	1 856	272	48 332	1 011	102,96	39 621
2413000	Powiat tarnogórski	2 194	12 526	10 921	711	84 801	2 804	105,70	71 853
2414000	Powiat bieruńsko-lędziński	965	13 558	3 128	117	37 535	559	102,86	30 301
2415000	Powiat wodzisławski	3 139	15 045	5 562	690	95 930	2 289	105,61	80 959
2416000	Powiat zawierciański	8 023	9 497	4 159	440	70 562	3 031	106,82	61 169
2417000	Powiat żywiecki	8 190	14 573	5 004	437	94 382	3 639	104,62	78 343
2461000	Powiat m.Bielsko-Biała	702	34 049	17 831	2 073	98 897	2 058	111,63	90 337
2462000	Powiat m.Bytom	335	7 839	6 875	1 161	100 912	4 406	109,42	87 149
2463000	Powiat m.Chorzów	171	8 217	7 338	780	64 674	1 878	110,29	56 871
2464000	Powiat m.Częstochowa	1 882	30 708	19 312	2 086	129 859	4 060	113,06	117 958
2465000	Powiat m.Dąbrowa Górnicza	399	22 724	8 121	934	71 168	2 878	108,97	62 710
2466000	Powiat m.Gliwice	656	34 312	19 517	1 975	106 697	3 371	108,00	93 361
2467000	Powiat m.Jastrzębie-Zdrój	1 737	14 547	3 506	927	53 437	1 487	104,79	45 607
2468000	Powiat m.Jaworzno	353	11 030	4 792	698	56 475	1 315	106,32	47 183
2469000	Powiat m.Katowice	606	34 294	47 268	14 433	174 719	3 579	110,24	154 427
2470000	Powiat m.Mysłowice	362	9 007	3 853	489	45 896	1 400	107,14	38 579
2471000	Powiat m.Piekary Śląskie	164	4 212	2 499	498	33 490	1 097	108,60	28 790
2472000	Powiat m.Ruda Śląska	221	15 324	5 751	2 167	83 904	1 275	107,03	71 343
2473000	Powiat m.Rybnik	685	17 701	9 163	1 027	84 254	1 936	105,00	71 038
2474000	Powiat m.Siemianowice Śląs	239	6 687	2 263	673	39 990	1 145	109,86	35 155
2475000	Powiat m.Sosnowiec	392	15 019	16 926	1 975	118 292	4 611	111,32	106 430
2476000	Powiat m.Świętochłowice	41	2 620	2 426	312	29 905	879	108,18	25 988
2477000	Powiat m.Tychy	262	25 542	8 885	1 103	75 889	1 594	107,69	66 281
2478000	Powiat m.Zabrze	219	12 342	8 222	1 756	106 680	3 110	107,31	89 744
2479000	Powiat m.Żory	733	7 236	3 069	448	37 196	671	104,64	31 936
2601000	Powiat buski	18 465	2 130	3 429	238	43 253	1 390	104,94	36 897

2602000	Powiat jędrzejowski	15 110	4 818	2 095	294	51 348	2 471	102,09	43 484
2603000	Powiat kazimierski	10 430	395	764	69	20 765	1 222	103,38	17 207
2604000	Powiat kielecki	26 430	8 135	5 286	244	133 446	7 957	100,56	105 643
2605000	Powiat konecki	6 523	5 891	2 046	250	48 626	3 405	103,13	40 946
2606000	Powiat opatowski	10 911	1 948	938	164	32 023	3 139	100,96	26 414
2607000	Powiat ostrowiecki	6 768	7 338	3 813	776	65 748	4 311	108,68	57 355
2608000	Powiat pińczowski	8 408	1 641	1 306	103	23 592	1 127	102,52	19 880
2609000	Powiat sandomierski	15 752	3 851	3 197	354	47 160	2 471	105,24	39 879
2610000	Powiat skarżyski	2 080	4 737	3 558	238	44 604	3 901	107,44	38 750
2611000	Powiat starachowicki	7 756	12 702	3 049	262	53 774	3 135	106,45	46 600
2612000	Powiat staszowski	11 204	6 442	1 794	277	44 366	2 265	102,15	36 468
2613000	Powiat włoszczowski	7 593	4 502	1 200	152	27 421	1 300	99,94	22 661
2661000	Powiat m.Kielce	1 389	19 903	19 573	2 967	114 411	6 024	113,37	104 021
2801000	Powiat bartoszycki	4 906	2 861	1 357	246	35 926	3 849	104,97	29 660
2802000	Powiat braniewski	2 806	1 199	1 359	166	26 427	2 863	100,11	20 707
2803000	Powiat działdowski	3 966	5 874	1 858	193	40 607	3 665	103,05	33 213
2804000	Powiat elbląski	4 171	4 903	1 576	129	36 051	3 183	99,95	28 768
2805000	Powiat ełcki	3 435	7 773	4 221	476	57 280	4 012	103,66	46 501
2806000	Powiat giżycki	3 239	2 717	1 794	238	35 150	1 577	104,46	28 996
2807000	Powiat iławski	5 663	12 391	3 144	338	57 109	1 699	102,01	46 973
2808000	Powiat kętrzyński	3 231	3 719	1 466	238	38 960	3 471	104,08	32 091
2809000	Powiat lidzbarski	2 912	2 582	1 146	118	25 713	1 970	102,85	21 048
2810000	Powiat mrągowski	2 975	3 546	2 290	220	31 588	1 973	104,24	25 560
2811000	Powiat nidzicki	1 650	2 915	934	103	20 641	879	101,33	16 643
2812000	Powiat nowomiejski	4 818	3 796	1 186	96	27 029	1 295	100,84	22 090
2813000	Powiat olecki	2 694	3 305	873	133	21 236	1 572	99,90	17 208
2814000	Powiat olsztyński	5 593	9 719	3 841	525	80 239	4 230	102,54	63 799
2815000	Powiat ostródzki	4 561	10 100	3 548	324	64 596	4 155	102,87	53 132
2816000	Powiat piski	2 852	2 386	1 397	203	35 523	2 019	100,65	28 377
2817000	Powiat szczycieński	4 841	5 113	1 850	320	43 932	2 116	102,16	35 316
2818000	Powiat gołdapski	2 204	1 977	491	90	16 679	940	101,21	13 535
2819000	Powiat węgorzewski	1 770	401	398	83	14 026	1 228	100,75	11 479
2861000	Powiat m.Elbląg	468	9 562	4 959	1 044	72 622	3 358	109,01	62 661
2862000	Powiat m.Olsztyn	475	14 151	16 280	3 002	103 001	3 087	115,14	92 244
3001000	Powiat chodzieski	2 523	6 889	1 433	180	28 787	1 326	103,56	24 027
3002000	Powiat czarnkowsko-trzcian	6 357	9 422	2 627	283	53 428	1 435	101,95	44 133
3003000	Powiat gnieźnieński	6 936	10 869	7 362	652	87 802	2 357	104,36	74 208

3004000	Powiat gostyński	7 617	9 796	2 642	496	46 782	1 816	102,03	38 387
3005000	Powiat grodziski	6 129	6 284	2 637	347	32163	943	100,89	26 101
3006000	Powiat jarociński	4 993	7 092	3 361	257	43 569	1 032	105,07	36 729
3007000	Powiat kaliski	15 492	4 522	2 460	128	51 245	742	103,44	42 217
3008000	Powiat kępiński	4 761	16 338	2 257	179	34 856	585	101,16	28 406
3009000	Powiat kolski	10 641	7 315	2 420	378	53 272	1 413	103,99	44 461
3010000	Powiat koniński	14 463	6 909	4 571	166	82 214	3 935	100,94	65 286
3011000	Powiat kościański	6 111	6 010	4 381	725	48 792	890	103,67	40 329
3012000	Powiat krotoszyński	7 402	10 014	5 028	235	47 252	1 161	103,44	39 400
3013000	Powiat leszczyński	6 121	7 324	2 549	175	34 853	693	100,44	28 304
3014000	Powiat międzychodzki	2 088	2 908	1 349	122	22 523	575	101,20	18 601
3015000	Powiat nowotomyski	4 263	12 417	3 231	339	45 820	688	103,74	38 373
3016000	Powiat obornicki	3 063	7 161	2 859	174	36 595	766	101,71	30 182
3017000	Powiat ostrowski	11 293	23 582	8 450	576	98 703	2 010	104,50	82 535
3018000	Powiat ostrzeszowski	6 559	6 655	1 500	138	34 052	1 067	102,53	28 047
3019000	Powiat pilski	6 439	13 017	8 525	945	83 787	2 429	104,41	69 784
3020000	Powiat pleszewski	6 999	4 877	2 597	220	38 619	895	101,70	31 839
3021000	Powiat poznański	9 251	54 492	51 625	1 457	238 933	2 620	105,30	200 194
3022000	Powiat rawicki	6 651	5 690	2 773	993	36 342	1 030	102,61	30 591
3023000	Powiat słupecki	6 496	3 779	1 613	293	36 684	1 753	100,95	29 826
3024000	Powiat szamotulski	5 126	13 509	4 028	363	55 705	1 181	104,03	46 493
3025000	Powiat średzki	3 620	6 493	3 248	230	34 173	1 391	102,99	29 658
3026000	Powiat śremski	3 925	5 994	2 812	621	37 413	445	103,59	31 203
3027000	Powiat turecki	9 259	10 692	4 128	276	51 753	1 637	104,21	42 918
3028000	Powiat wągrowiecki	5 076	5 463	2 155	201	43 131	1 360	101,34	35 371
3029000	Powiat wolsztyński	6 274	6 492	5 624	324	34 854	410	102,63	29 035
3030000	Powiat wrzesiński	5 116	13 710	3 698	270	47 319	1 154	104,66	39 717
3031000	Powiat złotowski	5 588	5 212	10 626	276	43 318	2 237	101,83	35 119
3061000	Powiat m.Kalisz	1 627	12 548	9 155	870	58 567	1 265	115,03	54 016
3062000	Powiat m.Konin	840	8 455	6 249	895	43 855	2 225	111,33	39 064
3063000	Powiat m.Leszno	451	8 582	5 454	947	37 688	1 187	108,63	33 298
3064000	Powiat m.Poznań	1 931	50 598	71 649	15 037	315 069	4 214	114,37	286 194
3201000	Powiat białogardzki	1 430	3 384	1 115	321	29 574	2 760	103,85	24 375
3202000	Powiat choszczeński	2 754	1 921	878	150	30 205	2 253	100,69	24 384
3203000	Powiat drawski	3 326	4 283	1 411	264	35 261	2 499	104,41	29 317
3204000	Powiat goleniowski	4 210	9 665	4 706	326	51 000	1 803	102,55	41 790
3205000	Powiat gryficki	2 515	2 006	2 294	260	37 708	1 672	101,90	30 593

3206000	Powiat gryfiński	3 099	3 578	2 980	336	51 221	2 397	101,97	41 668
3207000	Powiat kamieński	1 624	1 465	1 781	197	28 955	2 316	103,95	24 071
3208000	Powiat kołobrzeski	1 909	3 920	5 728	310	48 236	719	108,32	41 362
3209000	Powiat koszaliński	3 466	3 402	1 904	201	42 201	2 476	100,61	33 278
3210000	Powiat myśliborski	2 641	4 826	2 314	386	40 708	1 654	103,43	33 690
3211000	Powiat policki	1 520	10 433	7 896	208	50 154	1 387	103,97	40 512
3212000	Powiat pyrzycki	2 282	2 508	918	125	24 471	1 410	100,07	19 826
3213000	Powiat sławieński	3 146	2 732	1 840	182	34 686	2 540	103,62	28 706
3214000	Powiat stargardzki	3 858	8 762	4 802	719	73 640	2 950	104,13	61 272
3215000	Powiat szczecinecki	2 706	5 018	2 970	370	47 452	3 861	105,68	40 059
3216000	Powiat świdwiński	2 438	2 751	958	214	29 127	2 063	102,40	23 901
3217000	Powiat wałecki	2 096	3 442	1 715	253	32 884	1 385	104,91	27 372
3218000	Powiat łobeski	1 493	2 370	696	108	22 703	2 153	100,17	18 568
3261000	Powiat m.Koszalin	248	11 518	10 127	1 119	62 239	2 514	112,50	56 816
3262000	Powiat m.Szczecin	1 510	23 542	36 959	5 668	237 683	4 576	110,48	211 254
3263000	Powiat m.Świnoujście	175	1 697	3 137	414	24 549	614	107,77	21 220

Code	Powiat	Post-production age	Users of the sewage system	Users of the water supply	Users of the gas supply	Forest area	Green area others then forest	Cultural buildings adapted for the disable	Morality rate	Area
									death per 1000 persons	
		in persons	in persons	in persons	in persons	in hectares per capita	in hectares	no of buildings per 1 squere km		in squere km
0201000	Powiat bolesławiecki	19 022	74 306	88 698	44 104	0,852693	208,16	0,000144	10,60	1 304
0202000	Powiat dzierżoniowski	24 773	72 769	95 447	80 731	0,097363	466,38	0,000069	12,71	479
0203000	Powiat głogowski	19 779	79 683	87 692	65 529	0,108333	466,12	0,000101	9,53	443
0204000	Powiat górowski	7 127	15 513	34 282	13 090	0,600011	68,96	0,000085	11,58	738
0205000	Powiat jaworski	11 150	39 842	45 681	28 282	0,250927	233,35	0,000119	11,50	582
0206000	Powiat jeleniogórski	14 580	40 775	53 935	31 712	0,472318	230,58	0,000110	13,80	1 257
0207000	Powiat kamiennogórski	9 865	34 943	39 497	24 364	0,343081	51,56	0,000046	12,67	1 003
0208000	Powiat kłodzki	38 973	103 456	136 302	85 995	0,443208	653,88	0,000069	12,97	1 642
0209000	Powiat legnicki	11 198	41 602	53 602	20 957	0,209679	169,64	0,000163	11,39	744
0210000	Powiat lubański	12 400	35 342	46 359	25 686	0,184637	145,98	0,000091	12,34	428
0211000	Powiat lubiński	24 711	101 106	106 165	85 775	0,210495	492,74	0,000264	9,84	712
0212000	Powiat lwówecki	10 142	26 378	35 716	14 333	0,520569	101,54	0,000022	11,88	710
0213000	Powiat milicki	7 620	23 031	34 590	14 122	0,781386	224,36	0,000054	11,16	715
0214000	Powiat oleśnicki	21 679	70 925	104 766	47 146	0,306907	394,42	0,000084	9,81	1 049
0215000	Powiat oławski	16 114	59 949	72 810	42 457	0,133151	264,64	0,000039	9,19	524
0216000	Powiat polkowicki	11 516	54 535	62 071	39 062	0,449092	462	0,000302	10,11	779
0217000	Powiat strzeliński	9 225	21 091	42 044	11 508	0,123973	193,64	0,000160	11,48	622
0218000	Powiat średzki	10 640	27 500	53 854	11 994	0,165198	136,69	0,000037	10,28	704
0219000	Powiat świdnicki	36 166	116 488	152 124	102 146	0,064547	853,72	0,000102	11,70	741
0220000	Powiat trzebnicki	16 572	41 455	81 702	34 814	0,314377	263,28	0,000130	10,11	1 025
0221000	Powiat wałbrzyski	13 007	33 598	50 909	32 978	0,318709	203,14	0,000143	13,56	430
0222000	Powiat wołowski	10 123	32 329	45 559	23 606	0,497737	415,98	0,000085	11,28	675
0223000	Powiat wrocławski	23 269	92 657	142 086	65 547	0,081612	487,8	0,000048	8,12	1 118
0224000	Powiat ząbkowicki	15 051	35 154	59 225	25 067	0,244705	531,12	0,000107	13,24	802
0225000	Powiat zgorzelecki	20 283	63 333	85 355	33 830	0,444823	394,34	0,000111	12,87	839
0226000	Powiat złotoryjski	9 218	29 996	39 023	16 312	0,263515	107,11	0,000068	10,51	576
0261000	Powiat m.Jelenia Góra	22 291	71 130	77 396	71 873	0,044737	425,39	0,000025	13,54	109
0262000	Powiat m.Legnica	23 801	93 390	98 082	93 733	0,002338	547,86	0,000030	11,89	56
0264000	Powiat m.Wrocław	155 191	593 396	617 944	484 928	0,003487	4668,7	0,000019	11,08	293
0265000	Powiat m.Wałbrzych	29 655	92 479	109 682	104 433	0,022977	430,64	0,000018	14,24	85
0401000	Powiat aleksandrowski	12 016	30 605	52 019	8 519	0,063393	261,08	0,000090	13,28	475

0402000	Powiat brodnicki	14 125	49 502	74 665	2 571	0,288406	107,1	0,000063	10,41	1 040
0403000	Powiat bydgoski	19 482	65 483	107 684	26 511	0,482955	174,51	0,000196	8,36	1 395
0404000	Powiat chełmiński	9 888	32 426	51 161	16 942	0,068381	234,78	0,000077	9,77	527
0405000	Powiat golubsko-dobrzyński	8 414	27 863	43 709	2 917	0,269886	87,02	0,000066	10,88	613
0406000	Powiat grudziądzki	6 907	15 635	37 336	5 803	0,261484	87,78	0,000099	9,55	728
0407000	Powiat inowrocławski	34 210	114 329	158 667	71 162	0,078562	665,66	0,000025	11,93	1 225
0408000	Powiat lipnowski	11 951	23 802	63 128	217	0,349763	137,89	0,000091	11,61	1 016
0409000	Powiat mogileński	9 223	26 409	45 600	15 367	0,241505	47,22	0,000065	10,39	675
0410000	Powiat nakielski	15 986	45 548	81 206	22 831	0,298045	188,31	0,000219	10,03	1 120
0411000	Powiat radziejowski	8 547	16 180	39 540	1 425	0,073939	72,74	0,000123	12,26	607
0412000	Powiat rypiński	8 696	22 317	42 078	43	0,268558	99,96	0,000023	11,56	586
0413000	Powiat sępoleński	7 926	24 121	34 556	10 165	0,474882	118,98	0,000146	10,59	791
0414000	Powiat świecki	19 102	65 409	91 880	24 971	0,527743	246,48	0,000121	9,92	1 474
0415000	Powiat toruński	16 848	62 897	100 052	18 951	0,387676	87,19	0,000094	9,06	1 230
0416000	Powiat tucholski	9 071	34 997	45 304	13 107	1,082409	51,92	0,000165	9,87	1 075
0417000	Powiat wąbrzeski	6 853	19 100	32 910	7 121	0,122482	49,08	0,000029	10,12	502
0418000	Powiat włocławski	17 016	35 863	82 131	7 649	0,319085	105,87	0,000093	10,81	1 474
0419000	Powiat żniński	14 227	42 035	67 633	21 780	0,241261	281,64	0,000099	10,84	985
0461000	Powiat m.Bydgoszcz	88 750	326 169	339 581	281 557	0,013917	2970,38	0,000017	11,72	176
0462000	Powiat m.Grudziądz	22 265	87 452	91 328	84 430	0,011554	603,64	0,000021	13,23	58
0463000	Powiat m.Toruń	47 360	184 482	191 764	162 315	0,013931	940,09	0,000025	9,99	116
0464000	Powiat m.Włocławek	27 677	102 382	107 467	81 096	0,018624	312,2	0,000036	12,23	84
0601000	Powiat bialski	22 042	42 904	88 489	3 127	0,681875	125,91	0,000126	12,14	2 755
0602000	Powiat biłgorajski	21 227	60 018	96 824	17 575	0,646819	94,64	0,000049	10,75	1 681
0603000	Powiat chełmski	15 420	21 193	62 277	7 899	0,450842	245,57	0,000217	12,11	1 887
0604000	Powiat hrubieszowski	14 836	23 511	44 558	1 782	0,260158	151,5	0,000078	12,65	1 268
0605000	Powiat janowski	9 830	13 988	38 340	5 594	0,774681	40,38	0,000065	11,53	875
0606000	Powiat krasnostawski	15 971	22 390	55 183	20 526	0,247221	141,68	0,000141	13,29	1 031
0607000	Powiat kraśnicki	21 986	41 974	82 487	51 756	0,222678	300,16	0,000031	11,39	1 005
0608000	Powiat lubartowski	18 355	44 393	82 786	7 024	0,308403	199,82	0,000023	11,35	1 289
0609000	Powiat lubelski	28 870	35 168	124 926	67 105	0,112303	207,24	0,000039	9,94	1 680
0610000	Powiat łęczyński	10 019	29 971	55 871	25 044	0,152414	250,1	0,000139	9,09	637
0611000	Powiat łukowski	20 594	44 238	98 294	32 340	0,302621	145,98	0,000047	10,53	1 394
0612000	Powiat opolski	25 129	19 685	53 509	25 758	0,308764	139,04	0,000033	12,71	810
0613000	Powiat parczewski	7 753	15 637	30 374	501	0,677232	26,6	0,000143	13,00	952
0614000	Powiat puławski	27 657	69 667	104 501	67 557	0,210076	469,5	0,000044	11,06	934
0615000	Powiat radzyński	11 875	22 570	51 109	4 382	0,347388	52,16	0,000084	12,40	965

0616000	Powiat rycki	12 400	22 202	48 741	20 131	0,242565	138,26	0,000107	12,03	615
0617000	Powiat świdnicki	16 877	42 651	66 944	44 544	0,073349	404,47	0,000069	10,99	468
0618000	Powiat tomaszowski	18 453	32 064	68 325	23 799	0,390285	174,7	0,000060	11,82	1 489
0619000	Powiat włodawski	8 427	21 214	34 742	484	1,321688	73,62	0,000052	12,28	1 256
0620000	Powiat zamojski	23 033	18 089	61 736	25 410	0,407984	74,32	0,000094	12,46	1 870
0661000	Powiat m.Biała Podlaska	11 793	49 280	49 728	26 777	0,010812	166,92	0,000017	8,61	49
0662000	Powiat m.Chełm	15 067	58 478	60 898	39 806	0,004220	392,42	0,000032	10,65	35
0663000	Powiat m.Lublin	81 005	311 976	324 310	291 334	0,004839	2104,5	0,000029	10,47	147
0664000	Powiat m.Zamość	14 578	58 151	60 535	60 011	0,000788	436,12	0,000016	9,10	30
0801000	Powiat gorzowski	12 526	49 763	66 585	33 937	0,755998	322,31	0,000084	10,54	1 214
0802000	Powiat krośnieński	11 350	33 887	50 963	19 011	1,513701	179,76	0,000018	10,55	1 391
0803000	Powiat międzyrzecki	12 091	38 808	51 646	9 448	1,258523	276,76	0,000069	10,19	1 388
0804000	Powiat nowosolski	18 910	64 290	82 559	42 722	0,349093	278,86	0,000058	11,05	771
0805000	Powiat słubicki	8 646	33 675	43 711	21 097	0,998879	211,28	0,000106	9,89	999
0806000	Powiat strzelecko-drezdenec	10 094	27 369	45 887	12 721	1,270283	131,94	0,000081	11,67	1 248
0807000	Powiat sulęciński	6 825	23 224	32 586	4 766	1,849436	244	0,000142	11,40	1 178
0808000	Powiat świebodziński	11 182	47 134	52 477	30 514	0,706466	148,3	0,000054	11,04	937
0809000	Powiat zielonogórski	14 672	50 471	72 802	36 277	0,876828	355,92	0,000251	9,51	1 350
0810000	Powiat żagański	16 844	55 028	76 544	46 020	0,665176	326,88	0,000075	12,29	1 132
0811000	Powiat żarski	20 091	58 319	90 459	49 935	0,774472	1550,88	0,000062	11,95	1 393
0812000	Powiat wschowski	7 605	27 006	36 433	14 508	0,627533	174,76	0,000051	10,35	624
0861000	Powiat m.Gorzów Wielkopo	29 370	123 807	123 910	97 520	0,002969	877,52	0,000032	11,23	86
0862000	Powiat m.Zielona Góra	32 423	118 824	133 196	118 185	0,105369	567,72	0,000014	9,78	277
1001000	Powiat bełchatowski	21 291	75 474	106 827	50 015	0,250182	370,94	0,000106	9,15	968
1002000	Powiat kutnowski	23 514	57 735	92 337	4 937	0,046025	423,8	0,000082	14,54	887
1003000	Powiat łaski	11 057	21 325	42 937	1 298	0,270374	104,84	0,000060	11,93	618
1004000	Powiat łęczycki	11 547	18 153	47 365	1 484	0,088119	144,56	0,000040	13,81	773
1005000	Powiat łowicki	17 571	32 863	73 575	2 124	0,125021	110,36	0,000051	12,55	988
1006000	Powiat łódzki wschodni	14 763	27 733	68 316	32 023	0,169587	107,36	0,000070	11,10	500
1007000	Powiat opoczyński	15 555	49 491	70 806	12 172	0,423351	77,08	0,000065	11,91	1 040
1008000	Powiat pabianicki	28 979	80 619	111 550	71 286	0,109138	401,52	0,000034	12,95	492
1009000	Powiat pajęczański	11 153	22 380	49 972	870	0,411479	81,58	0,000078	11,66	804
1010000	Powiat piotrkowski	18 449	29 163	87 684	4 068	0,383911	135,2	0,000055	11,64	1 429
1011000	Powiat poddębicki	9 258	12 645	37 220	1 814	0,336370	162,13	0,000024	13,09	881
1012000	Powiat radomszczański	25 629	59 290	106 657	26 850	0,397063	206,26	0,000035	12,80	1 443
1013000	Powiat rawski	10 358	19 834	40 843	13 291	0,169268	249,16	0,000041	11,74	646
1014000	Powiat sieradzki	25 196	55 526	110 285	2 057	0,257206	402,68	0,000059	11,78	1 491

1015000	Powiat skierniewicki	7 853	4 865	33 722	57	0,431155	15,92	0,000026	12,52	753
1016000	Powiat tomaszowski	27 024	73 471	110 546	44 044	0,273856	340,35	0,000154	12,90	1 025
1017000	Powiat wieluński	16 461	36 237	73 088	1 130	0,297123	180,46	0,000222	11,71	926
1018000	Powiat wieruszowski	8 350	23 941	41 137	1 192	0,354954	89,64	0,000071	10,46	577
1019000	Powiat zduńskowolski	14 453	49 192	64 498	1 041	0,121337	227,62	0,000045	11,36	369
1020000	Powiat zgierski	38 425	106 516	159 450	59 024	0,097364	444,22	0,000036	12,12	855
1021000	Powiat brzeziński	6 783	13 243	29 653	69	0,157650	61,34	0,000065	12,55	359
1061000	Powiat m.Łódź	191 710	600 195	650 306	556 419	0,003829	3943,66	0,000016	14,56	293
1062000	Powiat m.Piotrków Trybuna	17 855	66 798	71 185	56 453	0,018791	309,6	0,000014	13,16	67
1063000	Powiat m.Skierniewice	10 970	42 839	44 968	32 336	0,003276	256,46	0,000083	10,27	35
1201000	Powiat bocheński	19 134	61 129	81 880	86 254	0,173364	134,86	0,000178	8,49	649
1202000	Powiat brzeski	16 933	33 995	69 787	69 786	0,118151	71,82	0,000139	9,02	591
1203000	Powiat chrzanowski	28 867	83 632	124 467	90 002	0,110210	607,61	0,000112	11,49	370
1204000	Powiat dąbrowski	10 907	24 952	52 610	36 830	0,101431	34,94	0,000051	9,58	530
1205000	Powiat gorlicki	21 027	62 317	44 558	75 872	0,383552	146,46	0,000119	9,44	966
1206000	Powiat krakowski	51 925	155 219	258 259	207 025	0,053935	297,82	0,000054	9,11	1 231
1207000	Powiat limanowski	20 374	49 192	68 466	68 133	0,279590	59,06	0,000023	7,91	951
1208000	Powiat miechowski	11 577	14 341	43 164	3 606	0,165281	72,82	0,000041	13,90	676
1209000	Powiat myślenicki	20 890	74 600	89 303	73 467	0,188089	73,68	0,000071	8,12	673
1210000	Powiat nowosądecki	33 106	98 579	119 786	106 344	0,315707	876,47	0,000046	7,89	1 549
1211000	Powiat nowotarski	33 740	116 452	93 759	19 891	0,285280	213,71	0,000068	8,37	1 474
1212000	Powiat olkuski	26 091	63 671	110 361	77 075	0,200495	268,03	0,000080	11,55	618
1213000	Powiat oświęcimski	33 621	99 507	150 853	123 049	0,025934	443,56	0,000091	10,68	406
1214000	Powiat proszowicki	9 161	15 076	40 059	6 728	0,017056	25,52	0,000046	11,12	415
1215000	Powiat suski	15 617	30 257	33 344	9 770	0,391175	47,62	0,000047	11,06	686
1216000	Powiat tarnowski	35 517	103 930	145 892	137 337	0,151388	99,62	0,000124	8,53	1 412
1217000	Powiat tatrzański	13 706	43 760	50 931	4 061	0,334368	110,18	0,000044	9,17	472
1218000	Powiat wadowicki	29 965	83 536	137 139	102 323	0,095194	209,16	0,000075	10,29	646
1219000	Powiat wielicki	21 972	61 200	114 450	109 396	0,051190	264,12	0,000071	8,41	411
1261000	Powiat m.Kraków	181 160	709 318	769 038	565 637	0,001762	4197,31	0,000043	10,18	327
1262000	Powiat m.Nowy Sącz	17 642	72 124	72 127	74 128	0,007495	251,32	0,000036	8,79	58
1263000	Powiat m.Tarnów	27 246	95 792	109 019	102 615	0,002533	277,36	0,000092	10,44	72
1401000	Powiat białobrzeski	6 235	9 604	25 107	8 576	0,480333	34,26	0,000030	11,54	639
1402000	Powiat ciechanowski	18 536	51 668	83 891	35 074	0,202219	296,31	0,000045	11,17	1 060
1403000	Powiat garwoliński	19 868	48 905	95 029	40 528	0,359346	181,68	0,000092	10,11	1 285
1404000	Powiat gostyniński	10 063	19 682	38 730	1 953	0,308417	120,62	0,000066	12,03	615
1405000	Powiat grodziski	18 660	58 067	85 659	63 007	0,045513	209,64	0,000106	10,39	367

1406000	Powiat grójecki	20 719	42 128	73 974	48 834	0,168695	171,28	0,000030	12,08	1 268
1407000	Powiat kozienicki	13 387	37 357	50 881	20 552	0,482827	116,8	0,000017	11,00	916
1408000	Powiat legionowski	22 863	82 357	83 238	85 304	0,101492	374,74	0,000060	9,09	390
1409000	Powiat lipski	7 994	8 129	26 153	3	0,462254	11,88	0,000029	13,90	740
1410000	Powiat łosicki	6 874	13 053	26 790	2 410	0,564385	33,68	0,000064	13,28	772
1411000	Powiat makowski	9 358	12 739	39 159	76	0,623103	32,3	0,000133	13,36	1 065
1412000	Powiat miński	29 451	83 772	138 236	52 071	0,165301	212,43	0,000039	10,25	1 164
1413000	Powiat mławski	14 245	34 581	67 190	30 119	0,340076	84,42	0,000027	12,08	1 182
1414000	Powiat nowodworski	6 848	44 155	68 718	22 288	0,228996	135,18	0,000050	11,81	695
1415000	Powiat ostrołęcki	14 594	24 980	64 988	9 387	0,754762	15,12	0,000068	9,47	2 092
1416000	Powiat ostrowski	15 267	29 849	65 186	4 559	0,482250	73,21	0,000041	11,61	1 218
1417000	Powiat otwocki	26 218	73 736	96 410	66 340	0,148684	168,47	0,000048	10,67	616
1418000	Powiat piaseczyński	33 294	133 774	175 527	124 928	0,061733	530,56	0,000076	9,15	621
1419000	Powiat płocki	20 901	35 790	99 331	8 824	0,279817	80,26	0,000099	11,44	1 796
1420000	Powiat płoński	17 847	30 780	80 213	24 386	0,223805	181,5	0,000034	11,62	1 380
1421000	Powiat pruszkowski	35 179	130 388	139 065	138 762	0,016639	487,48	0,000049	9,82	246
1422000	Powiat przasnyski	10 168	25 999	50 837	23	0,709981	95,78	0,000038	10,67	1 219
1423000	Powiat przysuski	8 887	14 855	37 910	26	0,586892	67,9	0,000072	13,32	801
1424000	Powiat pułtuski	10 059	22 111	45 765	9 703	0,305343	65	0,000000	11,67	827
1425000	Powiat radomski	27 001	60 829	138 160	42 783	0,269925	257,14	0,000046	9,69	1 530
1426000	Powiat siedlecki	15 413	31 019	72 476	8 695	0,375779	32,78	0,000148	10,84	1 603
1427000	Powiat sierpecki	10 237	21 545	49 600	3 152	0,224187	83,33	0,000019	12,42	852
1428000	Powiat sochaczewski	17 400	39 635	79 202	3 338	0,128743	96,84	0,000035	10,76	735
1429000	Powiat sokołowski	12 472	21 050	47 094	2 536	0,466167	65,78	0,000074	12,59	1 131
1430000	Powiat szydłowiecki	7 873	13 794	37 170	6 615	0,371585	218,96	0,000151	11,30	452
1432000	Powiat warszawski zachodn	23 377	77 478	99 548	88 254	0,115978	441,22	0,000077	9,80	534
1433000	Powiat węgrowski	13 884	23 914	57 544	2 612	0,516258	86,04	0,000030	11,84	1 221
1434000	Powiat wołomiński	41 168	168 562	185 032	176 361	0,112853	255,13	0,000037	9,00	954
1435000	Powiat wyszkowski	13 488	41 717	68 522	29 466	0,403007	89,77	0,000149	10,54	876
1436000	Powiat zwoleński	7 303	11 256	25 513	2 578	0,282301	49,2	0,000000	12,81	573
1437000	Powiat żuromiński	7 906	12 900	37 986	8	0,449038	59,04	0,000051	13,09	807
1438000	Powiat żyrardowski	16 535	46 288	69 419	34 318	0,158524	322,6	0,000040	12,97	533
1461000	Powiat m.Ostrołęka	11 275	47 812	49 393	42 031	0,009435	265,58	0,000057	8,65	34
1462000	Powiat m.Płock	28 794	108 876	115 734	74 593	0,003508	713,31	0,000017	11,30	88
1463000	Powiat m.Radom	49 424	195 281	204 079	169 730	0,003603	1273,02	0,000023	11,73	112
1464000	Powiat m.Siedlce	16 768	73 223	74 820	64 399	0,002663	227,5	0,000026	8,88	32
1465000	Powiat m. st. Warszawa	431 216	1 685 737	1 710 082	1 307 399	0,004013	8064,66	0,000035	11,12	517

1601000	Powiat brzeski	19 309	74 636	88 264	50 557	0,183121	328,88	0,000066	10,63	876
1602000	Powiat głubczycki	10 473	27 587	43 721	20 756	0,093883	143,92	0,000109	12,61	673
1603000	Powiat kędzierzyńsko-kozie	21 699	74 332	93 882	50 618	0,154213	268,64	0,000053	11,93	625
1604000	Powiat kluczborski	13 987	41 796	62 124	28 507	0,386194	287,46	0,000061	10,88	852
1605000	Powiat krapkowicki	13 526	45 362	63 189	24 764	0,164745	354,86	0,000235	10,50	442
1606000	Powiat namysłowski	8 898	26 024	41 146	14 652	0,487946	315,6	0,000281	11,00	748
1607000	Powiat nyski	31 302	97 591	129 637	65 806	0,116719	441,2	0,000073	12,03	1 224
1608000	Powiat oleski	13 831	30 306	61 241	8 184	0,531112	79,88	0,000294	10,47	973
1609000	Powiat opolski	13 285	94 259	120 948	18 355	0,568078	148,2	0,000105	9,51	1 534
1610000	Powiat prudnicki	12 222	29 947	53 197	23 757	0,116867	172,23	0,000126	11,97	572
1611000	Powiat strzelecki	15 410	57 121	73 765	15 474	0,405187	382,52	0,000147	9,89	744
1661000	Powiat m.Opole	31 725	121 458	124 584	94 373	0,011878	817,38	0,000016	9,87	149
1801000	Powiat bieszczadzki	4 234	10 647	15 619	202	3,662946	40,3	0,000183	8,96	1 139
1802000	Powiat brzozowski	12 466	37 439	14 876	51 677	0,253284	35,72	0,000061	9,65	539
1803000	Powiat dębicki	25 228	88 453	109 552	112 804	0,148863	142,6	0,000103	8,75	777
1804000	Powiat jarosławski	23 813	87 941	112 081	89 087	0,192527	141,72	0,000191	9,47	1 029
1805000	Powiat jasielski	22 763	67 175	54 461	98 017	0,274486	148,68	0,000035	9,56	831
1806000	Powiat kolbuszowski	11 222	37 341	57 080	39 051	0,446607	18,82	0,000160	8,56	774
1807000	Powiat krośnieński	22 021	37 341	57 554	98 226	0,343187	215,52	0,000107	9,41	993
1808000	Powiat leżajski	13 429	45 414	66 137	42 663	0,267150	128,81	0,000158	9,20	584
1809000	Powiat lubaczowski	11 084	41 749	52 907	22 788	1,139580	77,1	0,000305	10,95	1 308
1810000	Powiat łańcucki	15 791	66 742	76 211	61 139	0,116382	165,22	0,000161	9,12	452
1811000	Powiat mielecki	26 868	89 669	132 030	92 965	0,156316	484,36	0,000066	9,18	881
1812000	Powiat niżański	12 524	42 376	57 747	33 574	0,494774	94,72	0,000105	9,61	786
1813000	Powiat przemyski	13 258	47 235	44 509	38 873	0,653988	60,68	0,000067	8,86	1 211
1814000	Powiat przeworski	15 206	60 906	69 728	50 988	0,221012	189,56	0,000127	10,02	698
1815000	Powiat ropczycko-sędziszow	13 361	36 270	59 323	47 120	0,185793	46,04	0,000108	9,33	548
1816000	Powiat rzeszowski	30 409	122 859	136 146	112 013	0,162604	114,36	0,000094	8,67	1 153
1817000	Powiat sanocki	19 658	70 873	63 654	75 796	0,612338	198,2	0,000095	8,90	1 156
1818000	Powiat stalowowolski	23 766	72 544	101 442	80 907	0,397018	467,22	0,000122	9,12	832
1819000	Powiat strzyżowski	11 787	19 842	38 377	44 584	0,235675	33,72	0,000032	9,83	504
1820000	Powiat tarnobrzeski	10 806	35 698	51 649	38 402	0,335816	115,7	0,000131	9,84	521
1821000	Powiat leski	5 523	13 550	16 185	5 823	2,159529	60,28	0,000226	9,82	835
1861000	Powiat m.Krosno	11 455	42 086	43 936	45 064	0,000463	183,14	0,000043	9,41	44
1862000	Powiat m.Przemyśl	14 870	56 735	58 368	44 181	0,018762	248,66	0,000049	11,46	46
1863000	Powiat m.Rzeszów	39 404	183 262	187 838	173 493	0,001593	937,93	0,000047	7,83	120
1864000	Powiat m.Tarnobrzeg	11 315	41 358	46 282	44 018	0,013428	240,68	0,000043	9,53	85

2001000	Powiat augustowski	12 114	34 005	49 833	63	1,312298	133,18	0,000103	10,94	1 659
2002000	Powiat białostocki	28 975	90 129	131 975	30 939	0,793364	261,71	0,000081	10,67	2 975
2003000	Powiat bielski	13 617	28 483	49 906	111	0,550589	100,04	0,000055	15,21	1 385
2004000	Powiat grajewski	9 263	24 749	41 768	14	0,463661	78,25	0,000147	10,67	968
2005000	Powiat hajnowski	12 090	27 065	39 838	59	2,013316	71,11	0,000348	17,53	1 624
2006000	Powiat kolneński	7 049	12 649	30 937	0	0,545515	24,13	0,000234	10,97	940
2007000	Powiat łomżyński	9 353	10 453	41 913	858	0,607699	14,74	0,000098	11,43	1 355
2008000	Powiat moniecki	8 589	18 698	33 003	3	0,708797	62,3	0,000172	12,68	1 382
2009000	Powiat sejneński	4 397	5 326	15 464	0	1,787710	13,68	0,000199	12,25	855
2010000	Powiat siemiatycki	10 646	16 261	39 013	6 581	1,126970	161,84	0,000045	13,99	1 459
2011000	Powiat sokólski	14 938	28 523	54 009	6	0,782480	132,26	0,000104	14,02	2 055
2012000	Powiat suwalski	6 701	12 628	29 943	0	0,649418	15,94	0,000112	10,12	1 307
2013000	Powiat wysokomazowiecki	12 123	18 109	53 695	2 871	0,423881	73,84	0,000105	11,03	1 289
2014000	Powiat zambrowski	8 709	23 622	41 717	1 444	0,494906	74,54	0,000068	10,82	733
2061000	Powiat m.Białystok	62 960	286 466	289 956	268 009	0,006185	1190,03	0,000030	9,08	102
2062000	Powiat m.Łomża	12 827	59 325	61 310	13 757	0,000522	256,19	0,000032	8,35	33
2063000	Powiat m.Suwałki	12 862	66 149	67 276	14 715	0,012421	264,03	0,000029	8,61	66
2201000	Powiat bytowski	13 815	60 327	73 751	15 953	1,451945	210,4	0,000164	8,79	2 192
2202000	Powiat chojnicki	17 813	79 658	92 238	33 862	0,721044	437,42	0,000082	8,74	1 364
2203000	Powiat człuchowski	10 884	45 011	53 756	1 130	1,377122	725,18	0,000089	9,78	1 575
2204000	Powiat gdański	17 677	96 259	113 607	57 143	0,125565	279,02	0,000034	6,45	794
2205000	Powiat kartuski	18 938	79 405	132 070	28 225	0,252437	137,17	0,000037	7,10	1 121
2206000	Powiat kościerski	12 412	52 305	61 479	878	0,718052	131	0,000152	8,71	1 166
2207000	Powiat kwidzyński	14 762	62 596	79 946	42 524	0,230252	366,34	0,000132	9,33	835
2208000	Powiat lęborski	12 688	50 247	62 515	34 301	0,431144	270,82	0,000075	9,75	706
2209000	Powiat malborski	13 006	49 874	63 241	38 650	0,018360	334,3	0,000329	9,42	494
2210000	Powiat nowodworski	15 766	25 248	35 227	5 559	0,152153	129,95	0,000000	10,55	674
2211000	Powiat pucki	14 264	74 231	84 068	20 357	0,207041	209,59	0,000175	8,01	581
2212000	Powiat słupski	17 938	79 752	94 355	15 982	0,842012	527,02	0,000455	9,79	2 304
2213000	Powiat starogardzki	22 599	80 698	117 407	39 476	0,442793	207,98	0,000031	9,13	1 345
2214000	Powiat tczewski	21 945	99 587	113 141	61 434	0,087614	666,94	0,000043	9,82	697
2215000	Powiat wejherowski	35 106	171 631	203 631	96 189	0,259342	340,44	0,000056	8,32	1 285
2216000	Powiat sztumski	7 460	26 856	35 811	15 192	0,305015	147,4	0,000048	9,64	731
2261000	Powiat m.Gdańsk	112 174	450 136	466 631	351 718	0,010044	2021,25	0,000032	11,54	262
2262000	Powiat m.Gdynia	63 042	233 792	241 920	177 996	0,024189	738,11	0,000012	10,43	135
2263000	Powiat m.Słupsk	22 923	86 409	89 380	77 162	0,005232	322,74	0,000055	11,19	43
2264000	Powiat m.Sopot	11 343	36 035	36 036	30 616	0,024950	240,17	0,000028	12,61	17

2401000	Powiat będziński	36 999	101 843	148 361	96 558	0,051935	878,04	0,000034	13,32	364
2402000	Powiat bielski	32 382	89 267	141 747	130 179	0,076268	200,4	0,000109	9,73	459
2403000	Powiat cieszyński	37 568	110 237	146 087	132 774	0,155439	505,14	0,000090	10,90	730
2404000	Powiat częstochowski	28 837	59 030	123 271	30 149	0,326242	131,14	0,000082	12,20	1 521
2405000	Powiat gliwicki	23 721	75 983	114 951	53 628	0,186631	465,66	0,000095	10,93	664
2406000	Powiat kłobucki	17 767	45 592	80 994	799	0,308967	77,26	0,000071	11,48	889
2407000	Powiat lubliniecki	15 263	56 554	72 609	14 427	0,534635	264,17	0,000052	10,33	822
2408000	Powiat mikołowski	19 359	71 556	97 251	52 252	0,084680	304,31	0,000010	10,29	233
2409000	Powiat myszkowski	16 357	29 214	64 582	30 918	0,168682	68,62	0,000042	12,61	479
2410000	Powiat pszczyński	18 579	78 351	110 668	79 688	0,118794	560,66	0,000072	8,21	471
2411000	Powiat raciborski	22 812	62 639	106 472	50 477	0,122811	340,34	0,000092	11,35	544
2412000	Powiat rybnicki	14 646	34 787	76 440	20 215	0,092555	118,82	0,000154	9,35	224
2413000	Powiat tarnogórski	31 253	100 381	138 019	75 804	0,231118	1167,88	0,000064	10,64	644
2414000	Powiat bieruńsko-lędziński	10 537	54 732	59 326	30 699	0,038316	230,86	0,000100	8,66	158
2415000	Powiat wodzisławski	33 166	103 937	153 392	59 691	0,017511	487,1	0,000102	11,03	287
2416000	Powiat zawierciański	29 132	63 887	113 779	53 355	0,258256	397,7	0,000059	13,32	1 003
2417000	Powiat żywiecki	30 370	121 558	91 998	18 799	0,350735	266,12	0,000078	11,24	1 040
2461000	Powiat m.Bielsko-Biała	42 679	146 316	167 811	150 377	0,018027	532,54	0,000064	10,89	125
2462000	Powiat m.Bytom	39 450	166 778	166 777	135 816	0,008210	813,26	0,000012	12,66	69
2463000	Powiat m.Chorzów	25 138	79 372	108 405	86 663	0,002105	1416,74	0,000028	14,35	33
2464000	Powiat m.Częstochowa	58 488	196 124	214 154	162 752	0,002824	1131,38	0,000009	13,31	160
2465000	Powiat m.Dąbrowa Górnicza	30 286	106 521	119 347	92 146	0,038330	982,33	0,000042	12,06	189
2466000	Powiat m.Gliwice	43 656	162 620	175 780	147 241	0,008226	1014,51	0,000000	11,76	134
2467000	Powiat m.Jastrzębie-Zdrój	20 400	77 152	89 046	72 776	0,006516	491,72	0,000022	10,21	85
2468000	Powiat m.Jaworzno	20 252	80 685	90 445	32 019	0,061533	607,8	0,000066	12,32	153
2469000	Powiat m.Katowice	75 891	281 117	290 000	225 829	0,022204	1321,26	0,000058	12,22	165
2470000	Powiat m.Mysłowice	15 327	62 361	74 509	54 521	0,022390	200,52	0,000080	10,93	66
2471000	Powiat m.Piekary Śląskie	12 738	49 964	55 238	31 122	0,003849	183,94	0,000054	12,64	40
2472000	Powiat m.Ruda Śląska	29 589	122 363	136 695	96 625	0,011155	719,76	0,000043	11,47	78
2473000	Powiat m.Rybnik	29 125	109 618	134 868	80 332	0,032663	733,67	0,000043	10,14	148
2474000	Powiat m.Siemianowice Śląs	16 168	67 091	67 089	60 940	0,000550	510,4	0,000074	12,64	25
2475000	Powiat m.Sosnowiec	54 539	184 045	201 973	147 249	0,007112	1188,55	0,000020	12,78	91
2476000	Powiat m.Świętochłowice	11 675	49 953	49 953	36 874	0,043953	200,88	0,000040	13,27	13
2477000	Powiat m.Tychy	29 646	127 794	127 796	108 357	0,007173	977,69	0,000016	10,78	82
2478000	Powiat m.Zabrze	39 431	159 338	170 388	133 096	0,008751	809,68	0,000012	10,89	80
2479000	Powiat m.Żory	13 187	61 090	62 444	54 943	0,000737	487,22	0,000048	7,82	65
2601000	Powiat buski	17 092	37 229	65 406	28 145	0,147898	327	0,000069	12,24	968

2602000	Powiat jędrzejowski	19 418	32 948	66 436	706	0,285898	165,48	0,000035	12,08	1 160
2603000	Powiat kazimierski	8 056	10 419	27 043	30	0,036100	21,86	0,000089	12,15	422
2604000	Powiat kielecki	37 266	110 894	202 180	15 729	0,366951	91,86	0,000071	9,65	2 246
2605000	Powiat konecki	19 198	40 150	69 720	18 590	0,693811	180,66	0,000037	12,22	1 140
2606000	Powiat opatowski	11 925	18 378	43 827	13 334	0,270939	35,1	0,000057	14,77	911
2607000	Powiat ostrowiecki	27 820	75 515	104 713	64 829	0,172182	329,4	0,000036	12,80	617
2608000	Powiat pińczowski	9 531	20 262	36 177	731	0,280497	73,81	0,000102	12,70	613
2609000	Powiat sandomierski	18 061	29 242	68 133	42 022	0,061075	127,72	0,000090	12,78	676
2610000	Powiat skarżyski	19 288	54 096	72 132	46 074	0,310874	74,28	0,000040	13,38	395
2611000	Powiat starachowicki	22 137	65 987	86 417	51 557	0,261010	200,2	0,000100	12,21	523
2612000	Powiat staszowski	15 348	38 414	66 921	18 641	0,357289	173,32	0,000125	10,65	925
2613000	Powiat włoszczowski	9 985	23 178	37 936	228	0,852729	35,34	0,000088	12,20	908
2661000	Powiat m.Kielce	50 300	176 289	187 682	160 425	0,011705	782,6	0,000036	10,87	110
2801000	Powiat bartoszycki	11 929	39 908	55 294	24 805	0,552764	337,44	0,000086	11,57	1 307
2802000	Powiat braniewski	7 875	28 686	38 024	0	0,776806	165,1	0,000097	10,38	1 202
2803000	Powiat działdowski	12 114	43 305	61 015	31 184	0,421609	123,3	0,000076	11,56	954
2804000	Powiat elbląski	10 580	27 793	54 060	1 530	0,490140	131,57	0,000070	10,65	1 416
2805000	Powiat ełcki	16 175	72 861	86 609	42 003	0,271600	234,23	0,000044	9,05	1 113
2806000	Powiat giżycki	11 691	46 192	53 935	33 379	0,515530	124,96	0,000106	11,01	1 120
2807000	Powiat iławski	17 405	69 955	89 818	30 299	0,411019	261,9	0,000075	9,50	1 385
2808000	Powiat kętrzyński	13 283	45 295	59 627	31 645	0,328205	234,24	0,000222	11,80	1 213
2809000	Powiat lidzbarski	8 484	26 341	38 925	10 034	0,628672	61,86	0,000096	10,73	925
2810000	Powiat mrągowski	9 565	37 479	49 639	21 353	0,676747	191,72	0,000080	9,73	1 065
2811000	Powiat nidzicki	6 212	20 693	29 978	11 705	1,162805	161,54	0,000302	10,99	961
2812000	Powiat nowomiejski	7 896	21 193	40 475	13	0,332698	49,06	0,000045	10,36	694
2813000	Powiat olecki	6 579	23 384	30 217	12 204	0,680817	104,7	0,000087	10,31	874
2814000	Powiat olsztyński	21 861	83 143	115 263	39 693	0,878730	151,75	0,000087	9,52	2 837
2815000	Powiat ostródzki	20 557	76 309	100 350	41 241	0,521496	302,58	0,000067	11,46	1 766
2816000	Powiat piski	10 651	40 000	48 959	12 982	1,533160	123,52	0,000053	10,19	1 775
2817000	Powiat szczycieński	12 985	42 935	65 015	23 197	1,398341	78	0,000072	10,98	1 933
2818000	Powiat gołdapski	5 066	17 634	25 108	52	0,926266	36,23	0,000223	9,23	772
2819000	Powiat węgorzewski	5 024	13 584	22 404	9 865	0,636991	55,06	0,000044	12,64	693
2861000	Powiat m.Elbląg	27 782	113 707	118 616	102 834	0,017503	454,46	0,000017	11,36	80
2862000	Powiat m.Olsztyn	39 197	172 299	172 236	136 321	0,010823	876,44	0,000035	9,56	88
3001000	Powiat chodzieski	9 424	38 605	46 899	28 836	0,508148	114,98	0,000191	9,58	685
3002000	Powiat czarnkowsko-trzcian	16 912	53 963	80 165	14 986	1,051625	208,62	0,000092	10,04	1 806
3003000	Powiat gnieźnieński	28 652	111 426	141 272	73 588	0,125881	457,44	0,000034	10,21	1 255

3004000	Powiat gostyński	14 197	51 614	74 462	47 730	0,147461	275,72	0,000105	10,53	810
3005000	Powiat grodziski	8 614	29 276	49 433	32 154	0,292444	142,38	0,000019	9,43	642
3006000	Powiat jarociński	14 119	56 587	69 784	31 187	0,150442	204,12	0,000028	10,78	587
3007000	Powiat kaliski	15 828	28 914	78 729	10 978	0,284118	173,74	0,000048	10,34	396
3008000	Powiat kępiński	10 618	38 742	54 860	13 697	0,212230	254,8	0,000053	9,83	608
3009000	Powiat kolski	18 263	40 173	81 590	3 160	0,136278	189,18	0,000057	11,38	1 011
3010000	Powiat koniński	22 562	51 695	126 243	5 965	0,199236	201,98	0,000085	9,55	1 578
3011000	Powiat kościański	15 294	59 342	77 764	41 782	0,122721	318,16	0,000025	10,16	722
3012000	Powiat krotoszyński	14 983	45 432	75 475	38 308	0,170714	283,86	0,000039	10,12	714
3013000	Powiat leszczyński	9 391	33 358	53 534	14 806	0,351953	165,34	0,000159	8,90	806
3014000	Powiat międzychodzki	7 300	24 416	34 350	12 654	0,888826	114,12	0,000108	9,80	736
3015000	Powiat nowotomyski	13 787	45 435	69 833	42 623	0,513606	589,56	0,000080	9,79	1 014
3016000	Powiat obornicki	10 863	42 432	56 199	31 216	0,370138	167,88	0,000033	9,45	711
3017000	Powiat ostrowski	32 685	104 715	158 152	74 767	0,202426	511,82	0,000031	10,51	1 160
3018000	Powiat ostrzeszowski	10 638	31 217	53 650	15 471	0,485087	85,72	0,000036	10,58	773
3019000	Powiat pilski	27 359	112 998	131 103	76 700	0,266686	282,42	0,000081	10,08	1 268
3020000	Powiat pleszewski	12 251	33 064	60 132	6 600	0,217970	549,64	0,000095	10,15	713
3021000	Powiat poznański	62 414	303 049	374 761	291 618	0,108968	1049,89	0,000056	7,68	1 900
3022000	Powiat rawicki	11 890	40 991	58 424	31 219	0,136268	198,88	0,000033	10,11	554
3023000	Powiat słupecki	11 700	32 774	56 801	1 456	0,220786	206,82	0,000067	11,03	838
3024000	Powiat szamotulski	17 032	63 598	88 523	24 110	0,377551	432,28	0,000143	10,47	1 119
3025000	Powiat średzki	9 894	39 372	56 483	30 650	0,173735	384,4	0,000103	10,08	624
3026000	Powiat śremski	11 454	46 862	60 805	35 792	0,183283	637,08	0,000033	9,10	574
3027000	Powiat turecki	16 245	41 201	82 755	4 428	0,269237	451,78	0,000048	10,30	929
3028000	Powiat wągrowiecki	12 723	46 225	67 844	21 658	0,282391	161,57	0,000100	10,02	1 040
3029000	Powiat wolsztyński	10 452	43 806	52 647	31 995	0,359944	353,66	0,000122	8,84	680
3030000	Powiat wrzesiński	14 934	45 104	76 293	19 542	0,170364	555,51	0,000039	10,47	704
3031000	Powiat złotowski	12 885	49 494	62 893	8 898	1,111951	311	0,000115	10,57	1 660
3061000	Powiat m.Kalisz	25 515	90 923	98 432	70 711	0,004014	378,28	0,000010	12,86	69
3062000	Powiat m.Konin	18 876	69 029	72 262	24 455	0,003521	741,36	0,000054	10,92	82
3063000	Powiat m.Leszno	14 592	62 659	63 016	55 338	0,003775	235,1	0,000031	9,50	32
3064000	Powiat m.Poznań	131 335	509 378	527 925	416 193	0,007248	2969,9	0,000034	10,91	262
3201000	Powiat białogardzki	9 708	40 148	45 631	10 814	0,733787	186,44	0,000376	10,56	845
3202000	Powiat choszczeński	9 853	37 496	44 767	22 388	1,050246	272,17	0,000288	10,35	1 328
3203000	Powiat drawski	11 862	42 823	54 644	30 439	1,446661	192,78	0,000348	10,77	1 764
3204000	Powiat goleniowski	16 126	60 172	77 417	46 396	0,726208	323,58	0,000157	10,47	1 616
3205000	Powiat gryficki	12 146	40 328	56 453	34 977	0,345676	316,2	0,000445	10,87	1 017

3206000	Powiat gryfiński	16 565	56 230	77 557	25 076	0,771015	303,56	0,000073	10,95	1 870
3207000	Powiat kamieński	10 679	33 257	44 478	13 571	0,593947	177,2	0,000085	9,98	627
3208000	Powiat kołobrzeski	18 105	72 625	76 970	48 562	0,195411	637,85	0,000101	10,58	725
3209000	Powiat koszaliński	12 136	44 076	63 443	18 340	1,071820	153,36	0,000136	10,47	1 653
3210000	Powiat myśliborski	13 906	49 701	64 719	29 352	0,750924	427,52	0,000091	11,12	1 182
3211000	Powiat policki	13 210	72 892	79 056	59 868	0,287048	221,9	0,000164	7,34	665
3212000	Powiat pyrzycki	8 197	27 375	37 740	17 474	0,118764	179,52	0,000076	11,72	726
3213000	Powiat sławieński	11 459	35 504	53 912	23 603	0,527184	98,63	0,000035	10,44	1 043
3214000	Powiat stargardzki	25 013	98 594	116 740	70 799	0,312626	520,5	0,000025	10,03	1 520
3215000	Powiat szczecinecki	16 847	65 563	76 682	38 302	1,029329	822,46	0,000192	10,36	1 766
3216000	Powiat świdwiński	9 948	38 951	44 490	17 132	0,831582	209,4	0,000042	10,71	1 093
3217000	Powiat wałecki	11 064	40 522	52 018	25 580	1,455585	236,6	0,000187	10,25	1 415
3218000	Powiat łobeski	7 801	24 419	35 826	10 166	0,914173	307,9	0,000162	12,30	1 065
3261000	Powiat m.Koszalin	27 788	101 334	107 286	93 945	0,032013	508,56	0,000037	10,25	98
3262000	Powiat m.Szczecin	99 951	347 367	385 457	342 138	0,012656	1448,26	0,000010	12,29	301
3263000	Powiat m.Świnoujście	10 472	38 623	40 906	28 683	0,104706	340,74	0,000073	12,91	202
